# Evaluation of Cold Resistance in Alfalfa Varieties Based on Root Traits and Winter Survival in Horqin Sandy Land

**DOI:** 10.3390/biology13121042

**Published:** 2024-12-12

**Authors:** Tao Li, Tiexia Zhu, Zhongguo Liu, Ning Yang, Zhipeng Wang, Tiegang Yang, Kai Gao

**Affiliations:** 1 College of Grassland, Inner Mongolia Minzu University, Tongliao 028000, China; lit19@lzu.edu.cn (T.L.);; 2 Tongliao Meteorological Bureau, Tongliao 028000, China

**Keywords:** alfalfa, winter survival rate, root physiological characteristics, stoichiometric ratio, cold resistance, comprehensive evaluation, cluster analysis

## Abstract

The Horqin Sandy Land in China is a key alfalfa production base, challenged by low winter temperatures and large diurnal temperature shifts, affecting alfalfa’s winter survival. Alfalfa roots are the primary organs responsible for winter adaptability; consequently, by investigating the changes in the root physiology and nutritional components of alfalfa during the overwintering period, we can enhance our understanding of its mechanisms for cold resistance. Over the course of two years (2022–2023), field trials were conducted on 40 alfalfa varieties selected from both domestic and international sources for their potential cold resistance. This study assessed winter survival rates and analyzed root contents, including soluble sugars, starch, soluble proteins, and the concentrations of carbon (C), nitrogen (N), phosphorus (P) and their stoichiometric ratios. Principal component analysis, subordinate function analysis, and cluster analysis were employed for comprehensive evaluation. Biochemical markers varied significantly across varieties. The C, N, and starch contents in the roots were the main factors determining cold resistance. The varieties were categorized into four groups: Category I included five highly resistant varieties (‘Baimu 202’, ‘WL168HQ’, ‘Zhongmu No. 1’, ‘Gongnong No. 1’, and ‘Legacy’); Category II consisted of six moderately resistant varieties; Category III included twenty-eight slightly resistant varieties; and Category IV contained one non-resistant variety (‘3010’). This study recommends the adoption of the five varieties in Category I to enhance alfalfa cultivation in the Horqin region. This research provides valuable theoretical and practical guidance for improving the cultivation of alfalfa in the cold regions of northeastern China, supporting the development of the local livestock industry.

## Simple Summary

This study addresses the challenge of growing alfalfa in China’s Horqin Sandy Land, where low winter temperatures significantly affect the plant’s winter survival capacity. Alfalfa roots play a crucial role in winter survival. To understand how alfalfa withstands cold, researchers investigated root properties in 40 varieties over two years, focusing on nutrients like sugars, starch, and key elements. Findings showed that the levels of carbon, nitrogen, and starch in roots were key determinants of cold resistance. Based on these insights, alfalfa varieties were grouped by winter hardiness, with five top-performing varieties recommended for cultivation in Horqin. This research offers guidance for enhancing alfalfa production in cold areas, supporting the local livestock industry and agricultural sustainability in northeastern China.

## 1. Introduction

Alfalfa (*Medicago sativa*), often referred to as the ‘queen of forages’, is one of the most widely cultivated forage crops globally, valued for its high nutritional content and versatility [1]. It serves as a critical component of livestock feed due to its high protein content, digestibility, and palatability. Additionally, alfalfa provides ecological benefits, including reducing surface runoff through its extensive root system, contributing to soil and water conservation, and controlling wind erosion [2]. Its nitrogen-fixing ability further enhances soil fertility and promotes sustainable agricultural practices [3]. However, alfalfa is often cultivated in marginal environments in China, particularly in arid and semi-arid regions like Gansu, Ningxia, and Inner Mongolia [4]. These regions are primarily concentrated in northern China, where harsh winters challenge alfalfa survival, significantly affecting the yield and persistence of the alfalfa industry in China [5]. In Ladakh, alfalfa, an important forage crop, faces significant challenges due to cold and arid habitats. Increasing alfalfa cultivation is necessary to address the shortage of fodder for livestock [6]. Plant cold tolerance is closely associated with nutrient storage and physiological adaptation traits [7].

Plants’ winter survival is closely linked to plants’ cold resistance, which is closely tied to root systems’ resilience. Under cold conditions, plants activate cold-resistance mechanisms, a process termed cold acclimation [8,9]. Cold acclimation enhances the accumulation of endogenous and inducible components in plant cells. Endogenous components encompass metabolites with antioxidant activity, hormone-responsive metabolites, and osmoprotectants that limit ice nucleation and mitigate freeze-induced dehydration in plant cells [9,10,11]. Soluble sugars function as osmotic regulators by lowering the freezing point of cells through increased osmotic potential. They promote the accumulation of cold-protective substances such as proline, enhance membrane stability, and directly protect protoplasts, mitochondria, and sensitive coupling factors on membranes, thereby improving the overall cold resistance of plants [12]. Research has demonstrated that the soluble sugar content in alfalfa roots prior to winter is closely associated with cold resistance [13]. Starch, a carbohydrate, can be converted into soluble sugars under low-temperature conditions, enhancing osmotic regulation and increasing cold resistance [14]. Soluble proteins also function as osmotic regulators within cells; their levels increase under cold stress, thereby enhancing the plant’s cold tolerance [15].

The content of carbon (C), nitrogen (N), and phosphorus (P) in alfalfa roots, along with their stoichiometric ratios, plays a critical role in the physiological and ecological processes of plants, especially in relation to cold climates and winter survival. These elements are fundamental to plant growth and metabolism, with their stoichiometric ratios directly influencing roots’ physiological functions and stress resistance. Research suggests that a higher C content in roots during winter is generally correlated with increased cold resistance [16]. This correlation arises because the accumulation of carbohydrates, such as soluble sugars and starch, provides energy reserves for cells and safeguards cell structures against freezing damage under low-temperature conditions [17]. The CNP stoichiometric ratio reflects the plant’s strategies for resource acquisition and utilization and directly influences its survival capacity in cold environments [18,19,20]. Moreover, higher C/N and C/P ratios are often correlated with enhanced cold tolerance, indicating a strategy in which plants prioritize storing carbohydrates to meet energy demands in cold environments [21,22]. Modifying the stoichiometric ratios in roots can enhance the plant’s cold resistance and improve its winter survival rate, thereby playing a crucial role in agricultural production in cold regions.

The Horqin Sandy Land, a major livestock production base in Inner Mongolia, has experienced a steady increase in alfalfa cultivation, with a core production area currently spanning 50,000 hectares in Alukeerqin Banner, Chifeng City. This region experiences significant temperature fluctuations during winter, with the day–night temperature range in February ranging between −25 and 10 °C. The soil undergoes repeated freeze–thaw cycles, and the area’s low winter precipitation increases the risk of drought and frost damage [23], hindering the crop’s successful overwintering and significantly affecting the subsequent year’s production [24,25]. Consequently, alfalfa’s ability to survive the winter is a crucial factor limiting its production in the Horqin Sandy Land. Current research on alfalfa cultivation in the Horqin Sandy Land primarily addresses production performance, cutting times, and ecological impacts [26,27]. The mechanism of alfalfa overwintering in this region remains unclear, and it is uncertain whether the physiological and stoichiometric characteristics of the root system before and after overwintering are the key factors influencing alfalfa’s overwintering success. To address this gap, this study measured and analyzed the winter survival rates, the root contents of soluble sugars, starch, and soluble proteins, and the concentrations and stoichiometric ratios of C, N, and P across 40 alfalfa varieties from both domestic and international sources. A comprehensive evaluation was performed using principal component analysis (PCA), subordinate function analysis, and cluster analysis. The aim was to provide a theoretical foundation and technical support for the collection of drought-resistant alfalfa resources and the scientific selection of varieties suitable for cultivation in the Horqin Sandy Land.

## 2. Materials and Methods

### 2.1. Study Site

**Figure 1:**
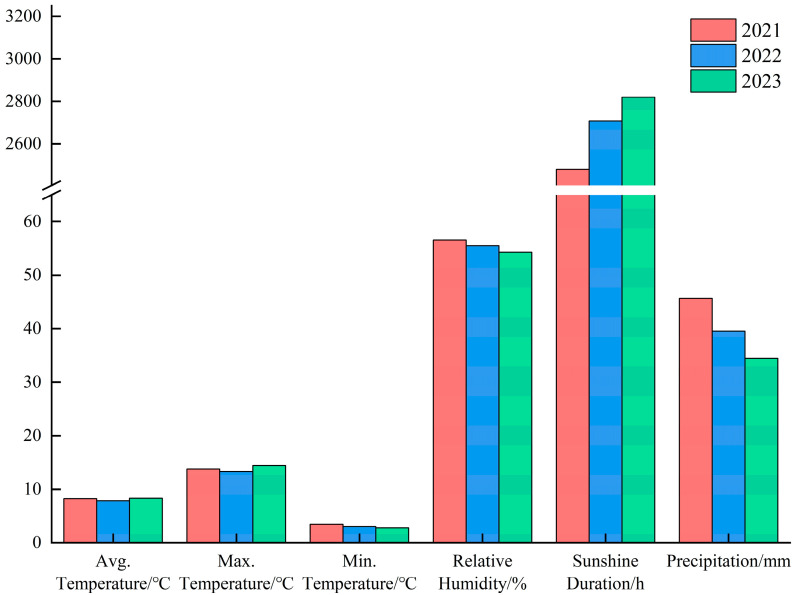
Meteorological data during the 2021–2023 experiment. The data in the figure are averages.

The experimental site is situated in the Science and Technology Demonstration Park of Inner Mongolia University for Nationalities, Tongliao, Inner Mongolia (43°36′ N, 122°22′ E). The site has an average annual temperature of 8.1 °C, with minimum temperatures ranging between −30 °C and −25 °C. The annual accumulated temperature exceeding 10 °C is 3137 °C·d. The number of days with temperatures below 0 °C varies between 86 and 98 days. The site receives an average of 2670 h of sunshine annually, with a frost-free period ranging between 135 and 145 days. The average annual precipitation is 39.86 mm, while the evaporation is 4.8 times greater. The seasonal average wind speed ranges between 3.1 and 4.3 m/s. This region is characterized by a typical temperate continental monsoon climate. The soil in the experimental field is sandy, with its basic characteristics detailed in Table 1. Meteorological data during 2021–2023 are shown in Figure 1.

### 2.2. Experimental Design

On 2 May 2021, forty alfalfa varieties were planted (Table 2), with each variety replicated three times, resulting in a total of 120 plots. Each plot covered an area of 6 m^2^ (3 m × 2 m). Seeds were sown in rows at a depth of 2 cm in sandy soil, with a seeding rate of 15 kg/hm^2^. Prior to sowing, nitrogen fertilizer (pure N) and potassium fertilizer (K_2_O) were applied as base fertilizers, each at a rate of 270 kg/hm^2^. The plots were mowed twice annually, with the first mowing on 13 July and the second on 28 August. After each mowing, nitrogen and potassium fertilizers were applied at a rate of 40 kg/hm^2^. Irrigation was performed every 5 days, with additional winter irrigation on 10 October to form a surface cover. Sprinkling was employed as the irrigation method, with a water application rate of 75 m^3^/hm^2^.

### 2.3. Root Sampling and Measurements

Before wintering (11 October) and after wintering (15 April), ten alfalfa plants exhibiting uniform growth were selected from each plot. A root auger with a diameter of 15 cm was specifically chosen for root sampling to ensure an adequate sampling volume, allowing for the collection of complete roots from alfalfa plants with deep and well-developed root systems from a depth of 0 to 40 cm. These samples were preserved at low temperatures and transported to the laboratory. The roots collected from each plot were mixed and then divided into two parts. One part was separated into taproots, root crowns, and lateral roots. The contents of soluble sugars, starch, and soluble proteins were immediately measured in these distinct parts. The other part was air-dried, ground into a fine powder, and utilized for measuring the concentrations of C, N, and P.

The soluble protein content was determined using the Coomassie Brilliant Blue method [28]. The soluble sugar and starch contents were measured using the anthrone colorimetric method [29].

The N and C content were determined with a Flash SMART elemental analyzer (Thermo Fisher Scientific, Bremen, Germany). The P content was measured with a fully automated high-tech discrete analyzer (Smartchem 450; AMS Alliance, Rome, Italy) following digestion with H_2_SO_4_-H_2_O_2_.

### 2.4. Winter Survival Rate

Winter Survival Rate (WSR): A 1 m section was randomly selected and marked within each plot, ensuring the avoidance of edge rows. The number of plants within the marked section was recorded. The following year, after alfalfa regreening, the number of surviving plants was counted to determine the winter survival rate (WSR):

WSR = (Number of surviving plants/Initial number of plants) × 100%.

### 2.5. Statistical Analysis

Statistical analyses were conducted using SPSS 21.0 software (SPSS Inc., Chicago, IL, USA). Data were analyzed using analysis of variance (ANOVA). Duncan’s method was employed to compare the means of the winter survival rate, root physiological characteristics, and chemometric characteristics among varieties at the *p* < 0.05 level. Pearson’s correlation analysis was conducted to determine the associations among different indicators. PCA, subordinate function analysis, and cluster analysis were used for comprehensive evaluation. Data plotting was performed using Origin 2023 software (Origin Inc., Northampton, MA, USA).

## 3. Results

### 3.1. Winter Survival Rates

The winter survival rates of the 40 tested alfalfa varieties varied significantly (Table 3), ranging between 13.00% and 93.67%. Varieties with winter survival rates exceeding 80.00% included ‘Zhaodong’ (93.67%), ‘Juneng 601’ (93.67%), ‘WL343HQ’ (91.33%), and ‘Gladiator’ (85.00%). The winter survival rates of ‘Zhaodong’ and ‘Juneng 601’ were significantly higher than those of all other varieties, except for ‘WL343HQ’ (91.33%) (*p* < 0.05). Varieties with winter survival rates below 40.00% included ‘WL656HQ’ (38.67%), ‘4030’ (36.33%), ‘Conthey’ (34.67%), ‘Juneng 995’ (32.67%), ‘Juneng S.R’ (32.33%), ‘3010’ (27.33%), and ‘Juneng 801’ (13.00%). Among them, ‘Juneng 801’ had a significantly lower winter survival rate than all other alfalfa varieties (*p* < 0.05).

### 3.2. Soluble Sugar Content

After measuring the soluble sugar content in the taproot, lateral roots, and root crown of different alfalfa varieties before and after winter, it was observed that the soluble sugar content in the roots showed a declining trend post-winter (Table 4). The variety ‘Jiasheng’ exhibited the largest decrease in the soluble sugar content in the taproot, with a reduction of 100.08 mg/g. The variety ‘Canon’ had the most significant decrease in the soluble sugar content in the lateral roots, with a reduction of 133.71 mg/g. The soluble sugar content in the root crown of the variety ‘WL656HQ’ decreased by 90.02 mg/g post-winter.

Before winter, the soluble sugar content in the taproots ranged from 67.20 to 139.14 mg/g. The variety ‘Canon’ had the highest soluble sugar content in the taproot (139.14 mg/g), which was significantly higher than that of 27 other varieties, excepting ‘Adina’, ‘Jiasheng’, ‘Baimu 202’, ‘WL168HQ’, ‘Zhaodong’, ‘4030’, ‘Legacy’, ‘Gladiator’, ‘Conthey’, ‘Challenger’, ‘Gongnong No. 1’, and ‘Knight 2’ (*p* < 0.05). The variety ‘WL525HQ’ had the lowest soluble sugar content in the taproot, at only 67.20 mg/g, which was significantly lower than that of all other varieties except ‘WL358HQ’ (*p* < 0.05). After winter, the soluble sugar content in the taproots ranged from 36.06 to 115.71 mg/g, with ‘Juneng 7’ having significantly higher levels than other varieties (*p* < 0.05). ‘Spider’ and ‘Juneng 401’ had the lowest soluble sugar content, at 36.06 mg/g and 37.19 mg/g, respectively, which were significantly lower than those of other varieties, excepting ‘Tecarat’, ‘Gongnong No. 1’, and ‘Jiasheng’ (*p* < 0.05).

Before winter, the soluble sugar content in the lateral roots of different alfalfa varieties ranged from 61.12 to 159.22 mg/g, with ‘Canon’ having significantly higher levels than other varieties (*p* < 0.05). ‘Juneng 401’, ‘4020’, and ‘Tango’ had lower soluble sugar contents, measuring 61.62, 64.94, and 66.44 mg/g, respectively, which were significantly lower than those of other varieties (*p* < 0.05). After winter, the soluble sugar content in the lateral roots of different alfalfa varieties ranged from 19.93 to 84.96 mg/g. ‘Challenger’ and ‘Spider’ had the highest soluble sugar contents, at 84.96 and 79.89 mg/g, respectively, which were significantly higher than those of other varieties (*p* < 0.05). ‘3010’ and ‘Canon’ had the lowest soluble sugar contents, measuring 19.93 and 22.51 mg/g, respectively, which were significantly lower than those of other varieties (*p* < 0.05).

Before winter, the variety ‘Adina’ had the highest soluble sugar content in the root crown (114.29 mg/g), while ‘Knight T’ had the lowest (56.34 mg/g). After winter, the soluble sugar content in the root crowns of different alfalfa varieties ranged from 16.97 to 86.77 mg/g. The variety ‘Challenger’ had the highest soluble sugar content (86.77 mg/g), which was significantly higher than that of the other varieties (*p* < 0.05). ‘WL656HQ’ and ‘Tecarat’ had the lowest soluble sugar contents, at 16.97 mg/g and 20.48 mg/g, respectively, which were significantly lower than those of the other varieties (*p* < 0.05).

### 3.3. Starch Content

After overwintering, the starch content in the roots of different alfalfa varieties showed a decreasing trend compared to that before overwintering (Table 5). The variety ‘Adina’ exhibited the largest decrease in the starch content in both the taproot and root crown, with reductions of 56.95 and 50.15 mg/g, respectively. The variety ‘WL525HQ’ had the most significant decrease in the starch content in the lateral roots, with a reduction of 74.33 mg/g.

Before winter, the starch content in the taproots of different alfalfa varieties ranged from 36.86 to 74.73 mg/g. The variety ‘WL440HQ’ had the highest starch content, which was significantly higher than that of all other varieties except ‘Juneng 995’, ‘Juneng 801’, ‘Adina’, ‘WL656HQ’, and ‘WL298HQ’ (*p* < 0.05). The variety ‘Tango’ had the lowest starch content in the taproot, which was significantly lower than that of all other varieties except ‘3010’ and ‘Spider’ (*p* < 0.05). After winter, the starch content in the taproots ranged from 12.47 to 55.70 mg/g. The highest and lowest starch content was found in the varieties ‘Gladiator’ and ‘Gongnong No. 1’, respectively.

Before winter, the starch content in the lateral roots of different alfalfa varieties was generally higher than in the taproots, ranging between 48.70 and 101.89 mg/g. The varieties ‘Juneng S.R’ and ‘Zhongmu No. 1’ had relatively high starch contents, which were significantly higher than those of the other varieties (*p* < 0.05). The variety ‘4020’ had the lowest starch content, which was significantly lower than that of all other varieties (*p* < 0.05). After winter, the starch content in the lateral roots of different alfalfa varieties did not differ significantly from that in the taproots, ranging between 14.54 and 54.06 mg/g. The variety ‘Tango’ had the highest starch content, which was significantly higher than that of the other varieties (*p* < 0.05). The varieties ‘3010’, ‘Fertress’, ‘Baimu 401’, ‘WL358HQ’, ‘WL525HQ’, ‘Gongnong No. 1’, ‘Zhaodong’, and ‘Canon’ had relatively low starch contents, which were significantly lower than those of all other varieties except ‘Milky Way’ (*p* < 0.05).

The starch content in the root crown of the alfalfa was lower than in the taproot and lateral roots. Before winter, the varieties ‘Juneng 801’ and ‘Conthey’ had relatively high starch contents in the root crown, measuring 72.15 and 66.99 mg/g, respectively, which were significantly higher than those of the other varieties (*p* < 0.05). The variety ‘Canon’ had the lowest starch content, which was significantly lower than that of all other varieties except ‘Juneng 401’, ‘4010’, and ‘Zhaodong’ (*p* < 0.05). After winter, the variety ‘Juneng 601’ had the highest starch content in the root crown (35.46 mg/g), which was significantly higher than that of all other varieties except ‘Challenger’ (32.89 mg/g) (*p* < 0.05). The variety ‘Adina’ had the lowest starch content (9.90 mg/g), which was significantly lower than that of all other varieties except ‘WL358HQ’, ‘WL525HQ’, and ‘Gongnong No. 1’ (*p* < 0.05).

### 3.4. Soluble Protein Content

The soluble protein content varied across the different root parts of the alfalfa (Table 6). Before winter, the soluble protein content was generally consistent in the taproot and lateral roots but was lower in the root crown. After winter, the differences in the soluble protein content among the three root parts were minimal. Similarly to the trends observed in the soluble sugar and starch content, the soluble protein content in the roots of different alfalfa varieties exhibited a declining trend after winter. The largest decreases in the soluble protein content were observed at 19.94 mg/g in the taproot, 23.81 mg/g in the lateral roots, and 16.72 mg/g in the root crown.

Before winter, the variety ‘WL298HQ’ had the highest soluble protein content in the taproot, at 29.54 mg/g, which was significantly higher than that of all other varieties except ‘WL168HQ’ (*p* < 0.05). The varieties ‘Tango’ and ‘Spider’ had lower soluble protein contents, at 13.37 and 13.52 mg/g, respectively, which were significantly lower than those of other varieties, excepting ‘4030’, ‘Juneng 995’, ‘Fertress’, and ‘Baimu 401’ (*p* < 0.05). After winter, the soluble protein content in the taproot decreased, ranging between 5.91 and 17.92 mg/g. The highest and lowest soluble protein contents were found in the varieties ‘Knight 2’ and ‘Adina’, respectively.

The soluble protein content in the lateral roots varied among the different alfalfa varieties. Before winter, the variety ‘WL168HQ’ had the highest soluble protein content in the lateral roots, at 35.62 mg/g, which was significantly higher than that of the other varieties (*p* < 0.05). The variety ‘4020’ had the lowest soluble protein content, at 14.16 mg/g. After winter, the soluble protein content in the lateral roots ranged from 5.80 to 14.04 mg/g. The highest and lowest soluble protein contents were found in the varieties ‘WL168HQ’ and ‘Tango’, respectively.

Before winter, the variety ‘WL168HQ’ had the highest soluble protein content in the root crown, reaching 26.39 mg/g, which was significantly higher than that of all other varieties (*p* < 0.05). The variety ‘4010’ had the lowest soluble protein content in the root crown, at only 10.79 mg/g. After winter, the variety ‘Knight 2’ had the highest soluble protein content in the root crown, at 12.60 mg/g, which represented a decrease of 6.05 mg/g compared to before winter. The variety ‘Zhaodong’ had the lowest soluble protein content, at only 5.07 mg/g, which represented a decrease of 9.31 mg/g compared to before winter.

### 3.5. C, N, and P Content

C, N, and P contents are vital indicators of winter survival in plants due to their roles in energy storage, protein synthesis, and metabolic regulation. Before winter, the C content in the roots of the different alfalfa varieties ranged from 32.59 to 56.08% (Table 7). The variety ‘Juneng S.R’ had the highest C content (56.08%), which was significantly higher than that of all other varieties except ‘3010’, ‘Juneng 995’, ‘4020’, ‘Tango’, ‘Fertress’, ‘WL358HQ’, and ‘Gongnong No. 1’ (*p* < 0.05). The variety ‘WL168HQ’ had the lowest C content, which was significantly lower than that of all other varieties except ‘WL343HQ’, ‘WL525HQ’, ‘WL298HQ’, and ‘Zhongmu No. 1’ (*p* < 0.05). After winter, the C content in the alfalfa roots slightly increased compared to before winter, ranging between 41.92 and 58.88%. Similarly to the trend before winter, ‘Juneng S.R’ and ‘WL168HQ’ remained the varieties with the highest and lowest root C contents, respectively.

Before winter, the N content in the roots of the different alfalfa varieties exhibited little variation. The varieties ‘Legacy’ and ‘4010’ had relatively high N contents, at 7.61% and 7.60%, respectively, which were significantly higher than those of ‘Conthey’, ‘Baimu 202’, ‘WL440HQ’, ‘WL168HQ’, ‘WL343HQ’, and ‘WL525HQ’ (*p* < 0.05). The variety ‘WL440HQ’ had the lowest N content, at 5.04%, which was significantly lower than that of ‘Juneng 995’, ‘4010’, ‘Legacy’, and ‘Canon’ (*p* < 0.05). After winter, the N content in the roots of different alfalfa varieties slightly decreased compared to before winter, ranging between 4.06% and 7.35%. The varieties ‘WL343HQ’, ‘Baimu 202’, ‘Juneng 401’, ‘WL440HQ’, ‘Tecarat’, and ‘Jiasheng’ had N contents greater than 7%, which were significantly higher than those of ‘Knight 7’, ‘Milky Way’, ‘Challenger’, ‘Adina’, ‘Conthey’, and ‘Greensilk’ (*p* < 0.05). The variety ‘Greensilk’ had the lowest N content, at only 4.06%.

The P content in the roots of the different alfalfa varieties showed little variation before and after winter, ranging between 0.20% and 0.42% before winter and from 0.18% to 0.36% after winter. Before winter, the variety ‘Gongnong No. 1’ had the highest root P content, which was not significantly different from that of ‘Spider’ and ‘Jiasheng’ (*p* > 0.05) but was significantly higher than that of the other 37 varieties (*p* < 0.05). The variety ‘Juneng 995’ had the lowest P content, which was not significantly different from that of ‘Juneng 401’, ‘Juneng S.R’, ‘Juneng 7’, ‘Juneng 801’, ‘Greensilk’, ‘Tecarat’, ‘WL656HQ’, and ‘WL440HQ’ (*p* > 0.05) but was significantly lower than that of the other 31 varieties (*p* < 0.05). After winter, the variety ‘Zhongmu No. 1’ had the highest root P content, significantly higher than that of all other varieties (*p* < 0.05). The varieties ‘Gladiator’ and ‘4020’ had lower P contents, at only 0.18% and 0.19%, respectively.

### 3.6. Stoichiometric Ratio

The stoichiometric ratios in the roots of different alfalfa varieties exhibited variation (Table 8). Before winter, the variety ‘WL440HQ’ had the highest C/N ratio at 9.59, while ‘WL298HQ’ and ‘Zhongmu No. 1’ had lower C/N ratios, at 5.45 and 5.54, respectively, which were significantly lower than those of ‘WL440HQ’, ‘Juneng S.R’, ‘Baimu 202’, and ‘Gongnong No. 1’ (*p* < 0.05). After winter, the C/N ratio in the alfalfa roots exhibited an increasing trend, ranging between 6.05 and 12.50. The highest and lowest C/N ratios were observed in the varieties ‘Greensilk’ and ‘WL168HQ’, respectively.

The N/P ratio in the roots of different alfalfa varieties showed little variation before and after winter, ranging between 15.53 and 37.31 before winter and from 17.05 to 37.55 after winter. Before winter, the variety ‘Juneng 995’ had the highest N/P ratio, which was significantly higher than that of 37 other varieties, excepting ‘Juneng 7’ and ‘Tecarat’ (*p* < 0.05). The variety ‘Zhaodong’ had the lowest N/P ratio, which was significantly different from that of ‘Juneng 401’, ‘Juneng 995’, ‘Juneng 7’, ‘Juneng 801’, ‘Tecarat’, and ‘WL656HQ’ (*p* < 0.05) but not significantly different from that of the other varieties (*p* > 0.05). After winter, the highest and lowest N/P ratios were observed in the varieties ‘WL343HQ’ and ‘Conthey’, respectively, with a significant difference between them (*p* < 0.05).

Before and after winter, the C/P ratio in the roots of the 40 tested alfalfa varieties exhibited an increasing trend. The highest and lowest values were observed in the same varieties, with ‘Juneng 995’ and ‘WL168HQ’ having maximum values of 264.26 and 292.98 and minimum values of 98.79 and 132.54, respectively. Before winter, the C/P ratio in the roots of ‘Juneng 995’ was significantly higher than that of the other 39 varieties (*p* < 0.05). After winter, the C/P ratio in ‘Juneng 995’ remained significantly higher than that of 35 other varieties, excepting ‘Juneng S.R’, ‘4020’, ‘Gladiator’, and ‘WL358HQ’ (*p* < 0.05).

### 3.7. Correlation Analysis

Correlation analysis revealed that the winter survival rate was significantly negatively correlated with the starch content in the taproot before winter (*p* < 0.05) and was highly significantly negatively correlated with the starch content in the root crown before winter (*p* < 0.01) (Table 9). The soluble sugar contents in the root crown before and after winter were highly significantly negatively correlated with each other (*p* < 0.01). The starch content in the taproot before winter was significantly positively correlated with the starch content in the root crown both before and after winter (*p* < 0.05). The starch content in the root crown after winter was highly significantly positively correlated with the starch content in the lateral roots both before and after winter (*p* < 0.01). The soluble protein content in the taproot before winter was highly significantly positively correlated with the soluble protein content in the lateral roots and root crown before winter (*p* < 0.01) and significantly positively correlated with the soluble protein content in the lateral roots after winter (*p* < 0.05). The soluble protein content in the taproot after winter was highly significantly negatively correlated with the soluble protein content in the lateral roots both before and after winter (*p* < 0.01).

The winter survival rate was highly significantly negatively correlated with the root C content after winter, the C/N ratio after winter, the N/P ratio before winter, and the C/P ratio before winter (*p* < 0.01), but it was highly significantly positively correlated with the P content before winter (*p* < 0.01) (Table 10). The root P content and C/P ratio were highly significantly positively correlated both before and after winter (*p* < 0.01). The root C content before winter was highly significantly positively correlated with the C/N and C/P ratios before winter (*p* < 0.01) and significantly positively correlated with the C/N and C/P ratios after winter (*p* < 0.05). The root C content after winter was highly significantly positively correlated with the C/N ratio before winter and with the C/P ratios both before and after winter (*p* < 0.01). The N content before winter was highly significantly negatively correlated with the C/N ratio before winter (*p* < 0.01) and highly significantly positively correlated with the N/P ratio before winter (*p* < 0.01). The root N content after winter was highly significantly negatively correlated with the C/N ratio after winter (*p* < 0.01) and highly significantly positively correlated with the N/P ratio after winter (*p* < 0.01). The root P content was negatively correlated with the N/P and C/P ratios, except for its non-significant correlation with the N/P ratio after winter (*p* > 0.05); it showed highly significant correlations with all other indicators (*p* < 0.01).

### 3.8. Comprehensive Evaluation

#### 3.8.1. Principal Component Analysis

**Figure 2:**
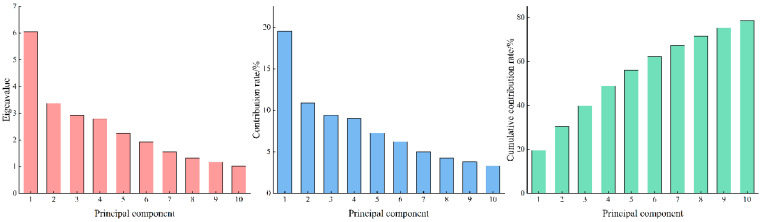
Variance contribution rate of different varieties of different alfalfa varieties.

PCA was conducted on the physiological indicators, CNP content, and stoichiometric characteristics of the roots of 40 alfalfa varieties before and after winter (Figure 2). Ten principal components were extracted, with eigenvalues of 6.051, 3.372, 2.918, 2.789, 2.253, 1.926, 1.546, 1.320, 1.171, and 1.025. The variance contribution rates for these components were 19.521%, 10.878%, 9.413%, 8.997%, 7.268%, 6.213%, 4.987%, 4.257%, 3.777%, and 3.306%. The cumulative variance contribution rate was 78.618%, indicating that the first 10 components sufficiently represented the majority of the information contained in the original variables.

Based on the calculation model by Wang et al. (2021), the data were substituted into the model to obtain 10 factors, labeled Y1~Y10. These 10 factors were then substituted into the equation “Y = (19.521 Y1 + 10.878 Y2 + 9.413 Y3 + 8.997 Y4 + 7.268 Y5 + 6.213 Y6 + 4.987 Y7 + 4.257 Y8 + 3.777 Y9 + 3.306 Y10)/78.618”, resulting in comprehensive scores for 40 different alfalfa varieties (Table 11). The top five varieties were ranked as follows: ‘Legacy’, ‘WL168HQ’, ‘Gongnong No. 1’, ‘Zhongmu No. 1’, and ‘Baimu 202’. The lower-ranked varieties included ‘3010’, ‘Quattro’, ‘Juneng 995’, ‘Juneng 401’, and ‘4020’.

#### 3.8.2. Subordinate Function Analysis

A comprehensive ranking of the 40 tested alfalfa varieties was performed using subordinate function analysis (Table 12). The top five varieties were ‘Juneng 601’, ‘Zhaodong’, ‘WL343HQ’, ‘Gladiator’, and ‘Fertress’, with comprehensive evaluation scores of 0.86, 0.84, 0.80, 0.79, and 0.76, respectively. The variety ‘Juneng 801’ had the lowest comprehensive evaluation score, at only 0.46.

### 3.9. Cluster Analysis

**Figure 3:**
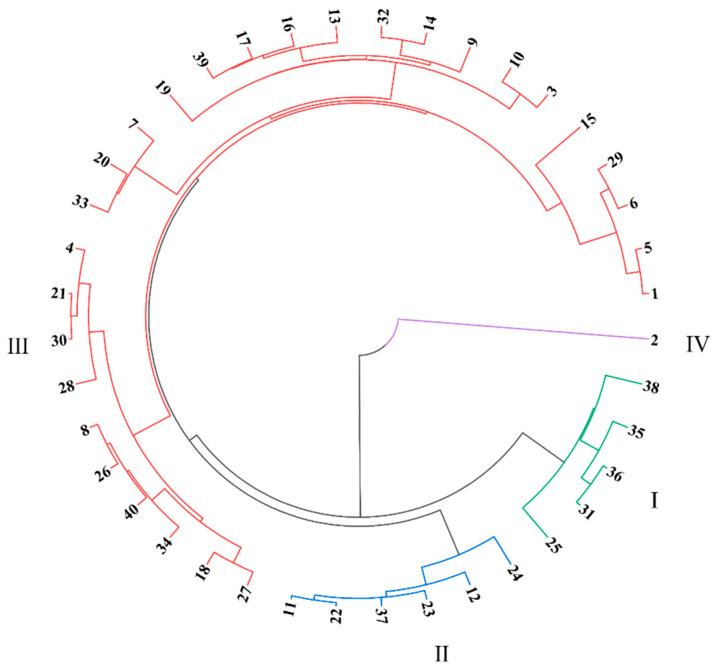
Cluster diagram of comprehensive scores based on principal component analysis and subordinate function analysis for 40 alfalfa germplasms. The numbers in the figure are the same as those in Table 1.

A systematic cluster analysis was performed on the 40 alfalfa germplasm varieties based on the comprehensive evaluation values obtained from the PCA and subordinate function analysis (Figure 3). The 40 alfalfa germplasm varieties were divided into four categories: Category I (green) included five germplasm varieties classified as highly cold-resistant, namely ‘Baimu 202’, ‘WL168HQ’, ‘Zhongmu No. 1’, ‘Gongnong No. 1’, and ‘Legacy’. Category II (blue) included six germplasm varieties classified as moderately cold-resistant. Category III (red) included twenty-eight germplasm varieties classified as slightly cold-resistant. Category IV (purple) included one germplasm variety (‘3010’) classified as non-cold-resistant.

## 4. Discussion

Low temperature is a significant abiotic stress factor that limits plant growth and development, thereby affecting crop yield and quality [30]. Alfalfa’s cold tolerance refers to its ability to overwinter successfully under low-temperature stress and regenerate in the following spring [31]. This encompasses both chilling and freezing stresses, involving two main processes: autumnal cold acclimation and winter freezing resistance. During the autumn cold acclimation phase, the above-ground parts cease growth, nutrients are transferred from the leaves and stems to the roots and root crowns, and a series of physical, biochemical, and molecular changes occur within the cells to prepare the plant for survival in the upcoming cold-resistance phase [32]. In the winter freezing phase, the aerial parts of alfalfa die off, and the plant relies primarily on its roots to withstand the cold. The physiological adaptability of the roots is crucial for successful overwintering. Consequently, this study evaluated and analyzed the winter survival rates of forty domestic and international alfalfa varieties, along with the root concentrations of soluble sugars, starch, soluble proteins, C, N, and P, and their stoichiometric ratios before and after overwintering. Comprehensive evaluations were conducted using PCA, subordinate function analysis, and cluster analysis. The results showed variations in the cold resistance among different alfalfa varieties, with physiological and nutrient content changes in the roots occurring by varying degrees before and after overwintering. Principal component and correlation analyses indicated that the contents of C, N, and starch in the roots were primary determinants of cold resistance in the alfalfa. Cluster analysis categorized the 40 alfalfa varieties into four groups. This research provides a scientific basis for selecting cold-tolerant alfalfa varieties and practical cultivation in China’s Horqin region, promoting the sustainable use of alfalfa grasslands in the area.

### 4.1. Winter Survival Rate

In the cultivation of alfalfa, the choice of variety plays a crucial role in determining its overwintering success and cold tolerance. Varietal performance in cold climates varies significantly, a phenomenon closely linked to physiological adaptability, genetic traits, and phenotypic characteristics. Phenotypic characteristics are key factors in the variability of winter survival rates in alfalfa. Some cold-tolerant varieties exhibit higher survival rates in cold environments, primarily due to deeper root systems [33]. Plants with deeper roots can access nutrients in subsoil layers, which are less susceptible to surface soil depletion. Deeper roots typically enhance plant resilience by providing stability against freezing-induced disturbances and preserving vital metabolic activities. The thermal buffering capacity of deeper soil layers shields roots from extreme surface temperatures, minimizing freeze damage to root tissues [34]. Furthermore, deeper root systems enhance carbohydrate storage and mobilization, supporting energy requirements during cold acclimation and recovery [35]. The genetic basis of different alfalfa varieties is diverse, particularly in the expression of cold-tolerance genes that regulate the plant’s cold acclimation and freezing response. This includes the regulation of antifreeze proteins, the synthesis of dehydration-protective substances (such as soluble sugars), and the increase in the unsaturation of cell membrane fatty acids [36]. Chen et al. further noted that cold-tolerant varieties enhance their tolerance to low-temperature stress by strengthening cell membrane stability and the expression of cold-adaptive proteins [37]. Cold-tolerant alfalfa varieties adapt more effectively to cold climates, enhancing their cold acclimation by accumulating more carbohydrates and antifreeze proteins in autumn to prepare for winter’s low temperatures. Moreover, after a harsh winter, these varieties show stronger bud recovery and earlier spring regreening, improving overall yield performance [33,38].

In this study, the 40 tested alfalfa varieties showed significant differences in winter survival rates. Varieties with winter survival rates exceeding 80% included ‘Zhaodong’ (93.67%), ‘Juneng 601’ (93.67%), ‘WL343HQ’ (91.33%), and ‘Gladiator’ (85.00%). ‘Zhaodong’ and ‘Juneng 601’ significantly outperformed all but ‘WL343HQ’. In alfalfa cultivation, selecting suitable cold-tolerant varieties significantly enhances winter survival rates and ensures early spring vigor and growth potential, laying a solid foundation for subsequent production.

### 4.2. Root Physiological Characteristics

Under cold stress, plant tissues exhibit an increase in soluble carbohydrate content. Soluble sugars, serving as cryoprotectants, function in osmotic regulation by providing energy reserves and lowering the freezing point through increased solute concentration in the cell sap. This process protects cells from freeze-induced solidification and dehydration while reducing membrane damage caused by ice formation [39,40,41,42,43]. Research indicates that leguminous forage roots accumulate substantial carbohydrates and soluble sugars prior to overwintering [44,45], consistent with this study’s findings. This study also observed a significant decline in soluble sugars in the roots of nearly all alfalfa varieties post-winter, likely due to these sugars being utilized for metabolic needs during overwintering and spring recovery. These results align with Dhont et al., who noted that plants gradually consume root sugars during winter to sustain metabolic functions and freezing tolerance [46]. Changes in sugar content also vary by root part. The taproot typically stores more nutrients and consumes them slowly during winter, while the lateral roots and root crown quickly utilize sugars in spring to support above-ground regeneration [47]. This difference is primarily due to the metabolic demands of each part; sugars in the taproot are slowly metabolized to maintain survival, while those in lateral roots are rapidly broken down to support regeneration [48].

Starch serves as an energy storage substance, providing essential energy for plants under low-temperature conditions and aiding their survival in cold environments. Studies indicate that a higher root starch content supports physiological processes crucial for cold resistance [49]. Before overwintering, forage roots increase starch accumulation to cope with low temperatures, ensuring adequate energy reserves for the winter season [13]. As overwintering progresses, starch is gradually broken down, supplying energy for the basic metabolic processes of the roots [50]. After overwintering, a notable decline in root starch content occurs, aligning with this study’s findings and the mechanism of plants consuming carbohydrates during winter to sustain metabolism and freezing tolerance. This study found significant differences in the starch content among different parts of the same variety before and after overwintering, indicating variations in starch storage and consumption within different root sections. Typically, the taproot undertakes long-term nutrient storage, while the lateral roots and root crowns are more involved in supporting spring regeneration, consistent with Regier et al.’s conclusions on functional zoning in alfalfa roots [48]. Substantial differences in starch consumption were observed among different varieties. For example, the taproot of ‘Juneng 401’ experienced a reduction of 33.90 mg/g, while its lateral roots’ consumption decreased by 9.72 mg/g. In contrast, the lateral roots of ‘Juneng 995’ exceeded the taproot in starch content after winter. This variation may have been related to the different functional roles of root parts. The taproot is primarily responsible for long-term storage and cold resistance, while the lateral roots and root crowns rapidly mobilize reserves to support spring growth [51].

The accumulation of soluble proteins in the root system is a key mechanism by which plants adapt to low-temperature environments, enhancing cold tolerance [49]. Under cold stress, alfalfa undergoes changes in soluble proteins and synthesizes antifreeze proteins. Research shows that an increase in soluble proteins is closely associated with enhanced cold tolerance, as protein levels rise when temperatures drop [52]. Sambe et al., confirmed that cold tolerance is positively correlated with the accumulation of cold-induced proteins [53]. Antifreeze proteins (AFPs) play a critical role in cold resistance by inhibiting ice crystal formation and recrystallization, which can damage cell structures [54]. Genes encoding AFPs, as well as cold-responsive (COR) genes, regulate the production of protective molecules, including sugars and stress-associated proteins [55]. During overwintering, some soluble proteins degrade to provide amino acids for sustaining metabolism [13]. This study found significant declines in soluble proteins in different alfalfa varieties after winter, with the greatest reductions in the taproot, lateral roots, and root crown being 19.94, 23.81, and 16.72 mg/g, respectively. Furthermore, post-winter variations in the protein content differed among root parts; for instance, the decline in soluble proteins in the taproot and lateral roots of ‘Juneng 995’ were 13.09 and 8.41 mg/g, respectively, with the lateral roots showing a more marked decrease. This suggests higher protein consumption in the lateral roots during overwintering, likely because the taproots are primarily used for energy storage while the lateral roots are more involved in metabolism and growth [51].

### 4.3. Root C, N, P Content and Stoichiometric Ratios

C, N, and P are essential elements for plant growth and development. C serves as the skeletal element of life, acting as the substrate and energy source for various physiological and biochemical processes, while N and P are crucial components of proteins and genetic materials and are limiting factors for plant growth. Genetic differences among various alfalfa varieties lead to variations in the accumulation of C, N, and P, consistent with the findings of this study. A higher C content is typically associated with greater overwintering capability, as stored carbohydrates provide an energy reserve that aids in cold resistance [56,57]. Additionally, C content can reduce ice crystal damage to cells through osmoregulation [13]. In this study, the C content in the roots of the alfalfa before overwintering ranged from 32.59 to 56.08% and increased to 41.92~58.88% after winter. This finding differs from Shahbaz et al. (2019) [58] and may be attributed to temperature increases that promote root growth, enhancing C accumulation to support the formation of new roots and the recovery of old ones. In this study, the varieties having a low C content before winter may have resulted from their metabolic prioritization of carbon utilization over storage during the growing season. Varieties like ‘WL168HQ’ could allocate a greater proportion of photosynthetically derived carbon towards growth and maintenance processes rather than storage in the form of starch or other reserves.

The N content in alfalfa roots during winter directly influences regeneration and greening in the following spring. Higher N levels before overwintering typically indicate a plant’s strong nutrient absorption and storage capabilities, enhancing its cold tolerance [57]. However, excessively high N levels may increase plants’ sensitivity to low temperatures, reducing overwintering success. During overwintering, N is heavily utilized in the synthesis of antifreeze proteins and other metabolic processes [59,60]. In this study, the N content in the roots of different alfalfa varieties after winter ranged from 4.06 to 7.35%, consistent with the findings mentioned above. Additionally, the reduction in N content varied significantly among different varieties; ‘Juneng 401’ experienced a decrease of 2.89 mg/g, while the N content in ‘Legacy’ decreased by only 1.24 mg/g. This may have been due to differences in N utilization efficiency among the varieties at low temperatures, with those with a higher efficiency better maintaining N reserves during overwintering.

Similarly to the pattern observed with the N, the P content in most varieties decreased after winter, consistent with Mahmood et al. [61]. P, a key element in energy metabolism (e.g., a component of ATP), supports energy transfer and cellular signaling at low temperatures [62]. Although the decrease in the P content before and after overwintering was slight, the difference remained, indicating relative stability during overwintering, sufficient to support spring regeneration.

The stoichiometric ratios of C, N, and P are critical characteristics of ecosystem processes and functions. The C/N ratios in plants reflect the dynamics of C accumulation and the patterns of N and P limitation within ecosystems. The N/P ratio can indicate whether plant growth is limited by the supply of N or P [63]. Tong et al. (2020) suggest that when the N/P ratio exceeds 16, plant growth is limited by P, and when it is below 14, growth is limited by N [64]. In this study, the N/P ratios in the roots of different alfalfa varieties ranged from 15.53 to 37.31 before overwintering and from 17.05 to 37.55 afterwards, showing an increasing trend. There was a significant negative correlation between the winter survival rate and root N/P before winter, indicating that P is a limiting element for alfalfa overwintering. In cold environments, P absorption is restricted, weakening the plant’s ability to regulate osmotic pressure at low temperatures, increasing the risk of freeze damage, and thus reducing the cold tolerance of alfalfa during overwintering. Higher C/N and C/P ratios are often associated with greater cold tolerance, reflecting the strategy of plants to store carbohydrates to meet energy needs in cold environments [20,21,22]. In this study, the C/P ratios ranged from 98.79 to 264.26 before winter and from 132.54 to 292.98 afterwards, also showing an increasing trend and a negative correlation with the winter survival rate. This further suggests that P limitation can prevent alfalfa from effectively using C to generate sufficient energy and metabolic substances to cope with the cold.

### 4.4. Comprehensive Evaluation

In germplasm comprehensive evaluation research, PCA, subordinate function analysis, and cluster analysis are widely used to reveal the genetic diversity and potential value of germplasm resources. The combination of these methods provides a systematic and comprehensive analytical framework, allowing researchers to effectively extract key information from high-dimensional data and categorize and evaluate germplasms. Prakash et al. (2023) employed PCA to quantify multiple traits in rice, identifying the main factors affecting yield and other critical traits [65]. This study, through correlation and PCA, found that the contents of C, N, and starch in the roots were the main determinants of cold resistance in the alfalfa. Carbon, a primary energy source for plants, particularly in cold environments, provides energy support for cold resistance responses and helps regulate intracellular osmotic pressure, reducing damage caused by cold. During overwintering, plants consume N to synthesize antifreeze proteins and other metabolites, enhancing cold tolerance [66]. Starch, a storage form of carbohydrates, is gradually hydrolyzed during overwintering to provide energy for plant metabolism and cold-resistance mechanisms. The negative correlation between pre-overwintering root starch content and the overwintering survival rate may be attributed to dynamic changes in starch metabolism and their impact on plants’ stress resistance [67]. Excessive starch accumulation may impede its conversion into soluble sugars, leading to a decline in cellular freezing tolerance [36]. This study conducted a comprehensive evaluation of 40 alfalfa varieties using PCA and subordinate function analysis, obtaining overall scores and rankings for each. Using these scores, systematic clustering was performed, dividing the 40 varieties into four groups: Group I included five germplasms that were highly cold-tolerant, including ‘Baimu 202’, ‘WL168HQ’, ‘Zhongmu No. 1’, ‘Gongnong No. 1’, and ‘Legacy’; Group II contained six moderately cold-tolerant germplasms; Group III comprised 28 slightly cold-tolerant germplasms; and Group IV included one germplasm (‘3010’) that was not cold-tolerant.

## 5. Conclusions

Scientific selection is a crucial step in agricultural production and breeding research, directly affecting crop yield and quality and contributing to local agricultural sustainability and environmental protection. This study focused on the cold tolerance of 40 alfalfa varieties collected both domestically and internationally in the Horqin Sand Land. It analyzed their winter survival rates, root soluble sugars, starch, soluble proteins, and C, N, and P contents, along with their stoichiometric ratios, using PCA, subordinate function analysis, and cluster analysis for a comprehensive evaluation. The results of this study not only enhance the theoretical framework of alfalfa’s cold resistance but also provide significant insights for selecting and breeding cold-tolerant alfalfa varieties in cold regions and establishes a theoretical basis for the sustainable development of alfalfa cultivation in northern China.

The results showed significant differences among the varieties in root soluble sugars, starch, soluble proteins, and C, N, and P contents, as well as their stoichiometric ratios. The principal component and correlation analyses indicated that the contents of C, N, and starch in the roots were the primary determinants of cold tolerance in the alfalfa. The cluster analysis based on scores from the principal component and subordinate function analysis grouped the 40 alfalfa varieties into four categories: Category I included five germplasms that were highly cold-tolerant, including ‘Baimu 202’, ‘WL168HQ’, ‘Zhongmu No. 1’, ‘Gongnong No. 1’, and ‘Legacy’; Category II contained six moderately cold-tolerant germplasms; Category III comprised 28 slightly cold-tolerant germplasms; and Category IV included one germplasm (‘3010’) that was not cold-tolerant. In conclusion, we recommend propagating the varieties ‘Baimu 202’, ‘WL168HQ’, ‘Zhongmu No. 1’, ‘Gongnong No. 1’, and ‘Legacy’ in the Horqin area due to their superior cold tolerance.

## Figures and Tables

**Table 1:** Basic chemical properties of soil in study site.

Soil Depth/cm	Alkali Hydrolyzed Nitrogen/(mg/kg)	Available Phosphorus/(mg/kg)	Available Potassium/(mg/kg)	Organic Matter/%	pH
0–10	11.28	4.58	104.90	4.88	7.00
10–20	11.15	4.47	92.46	4.81	7.05
20–30	10.98	4.41	87.45	4.75	7.15

**Table 2:** The names of the tested alfalfa varieties.

No.	Veriety	Fall Dormancy	Autumnal Dormancy Type	No.	Veriety	Fall Dormancy	Autumnal Dormancy Type
1	Juneng 401	4.2	H	21	Greensilk	2.0	A
2	3010	2.5	A	22	Challenger	2.5	A
3	4030	4.0	H	23	Adina	4.0	H
4	Juneng S.R	2.3	A	24	Conthey	3.0	A
5	Juneng 995	9.0	N	25	Baimu 202	2.0	A
6	4020	4.0	H	26	Baimu 401	4.0	H
7	Tango	—	—	27	Tecarat	7.0	N
8	Fertress	—	—	28	WL656HQ	9.3	N
9	Spider	1.0	A	29	WL358HQ	4.0	H
10	Gladiator	—	—	30	WL440HQ	5.0	H
11	Juneng 7	4.0	H	31	WL168HQ	2.0	A
12	Juneng 801	8.0	N	32	WL343HQ	4.0	H
13	Juneng 601	6.0	H	33	WL525HQ	8.0	N
14	4010	3.6	H	34	WL298HQ	3.0	A
15	Quattro	3.0	A	35	Zhongmu No. 1	2.0	A
16	Baimu 371	3.7	H	36	Gongnong No. 1	1.0	A
17	Baimu 341	3.4	H	37	Zhaodong	1.0	A
18	Knight 2	3.0	A	38	Legacy	—	—
19	Knight T	3.9	H	39	Canon	—	—
20	Milky way	4	H	40	Jiasheng	—	—

“—”, the autumn dormancy level information of this variety is unknown; A, autumnal; H, half-autumn sleeping type; N, non-autumn dormant type.

**Table 3:** Winter survival rate of different varieties of alfalfa (%).

No.	Variety	Winter Survival Rate	No.	Variety	Winter Survival Rate
1	Juneng 401	50.00 ± 1.57 o–r	21	Greensilk	60.67 ± 2.40 g–m
2	3010	27.33 ± 2.03 x	22	Challenger	52.33 ± 2.40 m–q
3	4030	36.33 ± 1.33 u–w	23	Adina	50.33 ± 2.40 n–r
4	Juneng S.R	32.33 ± 1.33 wx	24	Conthey	34.67 ± 2.19 v–x
5	Juneng 995	32.67 ± 1.45 wx	25	Baimu 202	66.67 ± 1.45 f–i
6	4020	69.00 ± 1.00 e–h	26	Baimu 401	69.33 ± 0.88 e–g
7	Tango	53.33 ± 2.19 k–q	27	Tecarat	66.67 ± 2.40 f–i
8	Fertress	79.67 ± 0.88 cd	28	WL656HQ	38.67 ± 2.03 t–w
9	Spider	59.67 ± 2.33 h–n	29	WL358HQ	55.00 ± 2.31 j–p
10	Gladiator	85.00 ± 2.00 bc	30	WL440HQ	43.00 ± 2.08 t–v
11	Juneng 7	74.33 ± 0.88 d–f	31	WL168HQ	62.00 ± 1.15 g–l
12	Juneng 801	13.00 ± 2.52 y	32	WL343HQ	91.33 ± 1.45 ab
13	Juneng 601	93.67 ± 1.20 a	33	WL525HQ	47.00 ± 2.08 p–t
14	4010	62.67 ± 1.86 g–k	34	WL298HQ	56.00 ± 1.73 j–p
15	Quattro	53.00 ± 1.15l m–q	35	Zhongmu No. 1	68.33 ± 2.03 e–h
16	Baimu 371	63.67 ± 3.18 g–j	36	Gongnong No. 1	76.00 ± 1.53 de
17	Baimu 341	60.33 ± 1.76 g–m	37	Zhaodong	93.67 ± 1.76 a
18	Knight 2	58.33 ± 1.86 i–o	38	Legacy	51.67 ± 0.67 m–r
19	Knight T	48.33 ± 2.03 p–s	39	Canon	40.00 ± 2.31 s–w
20	Milky way	68.00 ± 0.58 e–h	40	Jiasheng	45.00 ± 1.73 q–u

Note: The data in the table are averages. Different lowercase letters indicate significant differences between varieties. The same is in the table below.

**Table 4:** Soluble sugar content in roots of different varieties of alfalfa (mg/g).

No.	Variety	Soluble Sugar					
		Taproot		Lateral Root		Root Crown	
		Before Overwintering	After Overwintering	Before Overwintering	After Overwintering	Before Overwintering	After Overwintering
1	Juneng 401	111.84 ± 0.59 i–m	37.19 ± 1.53 m	61.62 ± 1.04 r	35.74 ± 2.00 mn	73.08 ± 1.13 f–l	65.13 ± 3.98 de
2	3010	91.86 ± 1.90 o–q	44.71 ± 0.66 i–k	76.35 ± 1.12 q	19.93 ± 1.03o	60.23 ± 1.71 lm	56.23 ± 1.89 f–h
3	4030	132.84 ± 1.09 a–e	60.05 ± 0.72 ef	103.19 ± 0.17 m	47.03 ± 1.10 h–k	71.76 ± 1.06 g–l	62.11 ± 1.67 d–f
4	Juneng S.R	113.06 ± 2.54 h–l	75.68 ± 1.69 bc	79.16 ± 0.79 q	61.61 ± 0.38 cd	85.81 ± 0.97 d–g	74.95 ± 0.73 bc
5	Juneng 995	114.05 ± 1.34 g–l	62.02 ± 0.25 e	80.53 ± 0.55 pq	44.22 ± 0.73 j–l	69.02 ± 1.20 h–m	55.36 ± 5.61 f–h
6	4020	102.60 ± 1.32 k–o	57.81 ± 0.86 ef	64.94 ± 1.05 r	44.54 ± 1.82 i–l	90.89 ± 1.57 b–e	42.89 ± 1.55 i–m
7	Tango	93.97 ± 1.37 n–q	76.32 ± 1.34 bc	66.44 ± 0.45 r	39.97 ± 1.39 k–n	79.10 ± 1.40 e–j	74.44 ± 1.35 bc
8	Fertress	123.60 ± 1.53 c–i	58.97 ± 1.03 ef	75.72 ± 0.83 q	43.08 ± 0.16 kl	74.80 ± 2.33 f–k	59.80 ± 1.36 ef
9	Spider	102.89 ± 1.75 k–o	36.06 ± 2.19 m	87.16 ± 0.70 o	79.89 ± 1.19 a	81.30 ± 1.23 d–i	68.85 ± 1.63 cd
10	Gladiator	128.54 ± 0.45 a–f	59.15 ± 0.52 ef	85.65 ± 1.07 op	60.30 ± 1.50 c–e	85.15 ± 2.34 d–g	62.72 ± 0.41 d–f
11	Juneng 7	87.21 ± 2.10 p–r	115.71 ± 1.38 a	119.59 ± 0.82 ij	58.28 ± 2.13 c–f	81.38 ± 2.72 d–i	59.48 ± 1.07 ef
12	Juneng 801	116.66 ± 0.55 f–k	62.00 ± 0.59 e	119.05 ± 0.28 ij	43.77 ± 0.82 j–l	100.68 ± 0.73 a–c	36.06 ± 0.86 m–o
13	Juneng 601	92.56 ± 0.50 o–q	53.63 ± 1.37 f–h	152.53 ± 1.77 b	34.81 ± 0.74 mn	73.43 ± 1.25 f–l	81.52 ± 1.76 ab
14	4010	110.14 ± 0.87 i–m	58.42 ± 1.33 ef	153.15 ± 0.55 b	43.89 ± 1.22 j–l	75.57 ± 1.66 f–k	44.96 ± 0.89 i–l
15	Quattro	111.56 ± 1.57 i–m	46.39 ± 1.41 i–k	137.47 ± 1.93 de	33.52 ± 1.73 n	67.42 ± 1.73 i–m	42.23 ± 0.98 i–n
16	Baimu 371	98.09 ± 0.67 m–p	58.17 ± 1.62 ef	149.30 ± 0.52 bc	59.28 ± 1.56 c–e	81.77 ± 1.78 d–i	41.04 ± 1.36 j–n
17	Baimu 341	117.93 ± 1.69 f–j	71.85 ± 1.49 cd	124.05 ± 1.17 g–i	57.83 ± 0.81 c–f	61.64 ± 1.48 k–m	61.95 ± 1.31 d–f
18	Knight 2	126.18 ± 1.48 a–h	72.59 ± 0.29 cd	134.32 ± 2.67 d–f	54.75 ± 1.21 d–g	64.13 ± 2.26 k–m	68.83 ± 0.88 cd
19	Knight T	106.73 ± 1.88 j–n	44.64 ± 0.79 i–k	126.87 ± 0.66 gh	61.03 ± 1.82 cd	56.34 ± 2.04 m	68.44 ± 1.33 cd
20	Milky way	124.21 ± 2.88 b–i	44.91 ± 0.90 i–k	94.94 ± 1.19 n	33.41 ± 1.68 n	56.72 ± 0.77 m	42.67 ± 1.70 i–m
21	Greensilk	103.41 ± 0.23 k–o	78.82 ± 1.12 b	123.61 ± 0.35 hi	61.12 ± 0.95 cd	73.26 ± 13.17 f–l	43.38 ± 0.97 i–m
22	Challenger	126.84 ± 13.32 a–h	78.62 ± 2.14 b	140.03 ± 0.74 d	84.96 ± 1.33 a	92.47 ± 2.10 b–e	86.77 ± 1.38 a
23	Adina	138.82 ± 1.49 a	68.78 ± 0.34 d	133.53 ± 1.62 ef	50.30 ± 1.07 g–j	114.29 ± 1.88 a	35.88 ± 0.62 m–o
24	Conthey	127.73 ± 2.58 a–g	57.74 ± 1.24 ef	119.83 ± 1.01 ij	54.57 ± 1.78 d–g	112.33 ± 2.32 a	44.02 ± 0.70 i–m
25	Baimu 202	136.29 ± 1.60 a–c	76.11 ± 1.37 bc	99.07 ± 1.99 mn	72.17 ± 1.10 b	66.21 ± 1.96 j–m	50.14 ± 2.15 g–i
26	Baimu 401	119.00 ± 2.36 e–j	55.52 ± 1.47 e–g	129.33 ± 0.29 fg	51.24 ± 1.81 f–i	87.36 ± 1.80 c–f	39.28 ± 0.67 k–o
27	Tecarat	121.81 ± 0.57 d–i	38.43 ± 1.28 lm	136.48 ± 1.57 de	46.56 ± 0.78 h–l	73.42 ± 1.26 f–l	20.48 ± 1.14 p
28	WL656HQ	101.86 ± 2.06 l–o	58.70 ± 0.94 ef	96.31 ± 1.01 n	57.43 ± 1.88 c–f	106.99 ± 1.14 a	16.97 ± 1.23 p
29	WL358HQ	75.99 ± 0.86 rs	56.40 ± 1.12 e–g	137.41 ± 1.38 de	63.04 ± 1.82 c	80.23 ± 1.97 d–j	31.58 ± 0.83 o
30	WL440HQ	101.56 ± 2.19 l–o	60.70 ± 0.31 e	146.59 ± 2.59 c	71.40 ± 1.05 b	84.21 ± 2.34 d–g	50.42 ± 1.05 g–i
31	WL168HQ	134.46 ± 2.55 a–d	69.36 ± 1.23 d	115.90 ± 0.98 jk	43.06 ± 1.50 kl	82.48 ± 2.05 d–h	49.34 ± 0.63 h–j
32	WL343HQ	84.05 ± 2.28 qr	58.16 ± 1.64 ef	115.59 ± 0.84 jk	60.12 ± 2.24 c–e	93.71 ± 2.63 b–d	46.71 ± 1.32 i–k
33	WL525HQ	67.20 ± 1.69 s	50.44 ± 1.73 g–i	135.00 ± 1.49 d–f	39.54 ± 0.89 l–n	106.70 ± 1.76 a	42.33 ± 1.18 i–m
34	WL298HQ	100.33 ± 2.26 l–p	54.10 ± 0.72 f–h	103.16 ± 1.25 m	53.79 ± 1.40 e–g	101.28 ± 0.47 ab	57.43 ± 1.66 e–g
35	Zhongmu No. 1	111.85 ± 2.49 i–m	49.28 ± 1.48 h–j	98.60 ± 1.20 mn	51.94 ± 1.15 f–h	110.80 ± 1.66 a	33.88 ± 1.53 no
36	Gongnong No. 1	126.49 ± 1.21 a–h	41.54 ± 1.28 k–m	138.97 ± 1.66 de	56.38 ± 1.52 c–g	110.06 ± 2.06 a	35.64 ± 1.50 m–o
37	Zhaodong	134.13 ± 1.54 a–d	43.66 ± 0.71 j–l	111.30 ± 1.47 kl	40.86 ± 0.97 k–m	87.22 ± 0.72 c–f	41.17 ± 1.40 j–n
38	Legacy	128.97 ± 1.27 a–f	69.28 ± 1.73 d	108.89 ± 0.36 l	72.54 ± 1.82 b	81.66 ± 2.26 d–i	48.55 ± 0.43 h–j
39	Canon	139.14 ± 3.01 a	48.23 ± 1.35 h–j	159.22 ± 0.20 a	25.51 ± 0.77 o	85.99 ± 1.05 d–g	37.80 ± 0.77 l–o
40	Jiasheng	138.19 ± 2.13 ab	38.11 ± 0.94 lm	120.66 ± 0.85 ij	44.92 ± 0.99 i–l	93.51 ± 2.00 b–e	46.44 ± 1.10 i–k

**Table 5:** Starch content in roots of different varieties of alfalfa (mg/g).

No.	Variety	Starch					
		Taproot		Lateral Root		Root Crown	
		Before Overwintering	After Overwintering	Before Overwintering	After Overwintering	Before Overwintering	After Overwintering
1	Juneng 401	61.15 ± 0.87 c–f	27.25 ± 0.35 h–l	70.97 ± 1.09 qr	22.64 ± 0.74 kl	25.82 ± 0.61 lm	23.27 ± 1.11 e–i
2	3010	38.21 ± 1.08 no	19.80 ± 0.83 p–r	64.12 ± 1.20 st	14.94 ± 1.54 m	43.18 ± 1.78 f–h	20.29 ± 0.61 h–m
3	4030	48.83 ± 1.68 j–l	27.99 ± 0.61 h–k	85.73 ± 0.92 b–e	23.40 ± 1.70 j–l	43.34 ± 0.96 f–h	20.61 ± 0.73 h–m
4	Juneng S.R	58.97 ± 0.66 d–g	36.52 ± 1.36 bc	100.04 ± 0.67 a	26.17 ± 0.79 h–k	45.99 ± 1.61 e–g	30.30 ± 1.84 bc
5	Juneng 995	72.06 ± 0.89 ab	24.85 ± 0.18 j–o	67.96 ± 1.76 rs	32.75 ± 0.98 c–e	54.39 ± 0.61 b–d	16.69 ± 0.72 l–p
6	4020	50.00 ± 1.29 i–l	24.79 ± 0.98 j–o	48.70 ± 0.48 v	48.64 ± 1.05 b	45.87 ± 2.43 e–g	21.70 ± 1.01 h–k
7	Tango	36.86 ± 1.10 o	28.89 ± 0.53 g–j	66.14 ± 0.73 s	54.06 ± 1.24 a	44.56 ± 1.90 e–g	26.68 ± 0.69 c–f
8	Fertress	46.64 ± 1.37 k–m	24.37 ± 1.16 k–o	73.43 ± 0.64 n–q	16.49 ± 1.13 m	49.57 ± 1.47 c–f	18.85 ± 1.13 i–n
9	Spider	41.02 ± 0.63 m–o	21.75 ± 0.66 m–p	83.49 ± 1.27 c–g	35.85 ± 1.88 cd	47.01 ± 0.66 d–f	22.49 ± 0.70 f–i
10	Gladiator	57.97 ± 1.21 e–h	55.70 ± 1.72 a	67.02 ± 0.15 rs	26.91 ± 1.66 g–k	56.24 ± 1.16 bc	28.93 ± 1.27 b–d
11	Juneng 7	59.81 ± 1.40 d–f	26.10 ± 0.68 i–m	85.38 ± 1.59 b–f	29.81 ± 0.22 e–h	51.38 ± 1.20 c–e	21.93 ± 0.78 g–j
12	Juneng 801	69.84 ± 1.43 ab	32.15 ± 1.04 d–g	88.37 ± 0.25 b	32.34 ± 1.45 c–f	72.15 ± 1.28 a	22.24 ± 0.82 f–j
13	Juneng 601	57.89 ± 1.17 e–h	31.29 ± 0.60 e–h	88.45 ± 0.94 b	25.75 ± 0.78 h–k	35.26 ± 1.06 ij	35.46 ± 0.75 a
14	4010	49.99 ± 2.12 i–l	26.03 ± 0.70 i–m	72.71 ± 0.20 pq	33.39 ± 0.96 c–e	27.30 ± 1.08 k–m	22.92 ± 1.34 e–i
15	Quattro	54.12 ± 1.35 f–j	32.33 ± 0.29 c–g	73.28 ± 0.65 o–q	32.60 ± 1.08 c–f	35.16 ± 1.56 ij	21.23 ± 1.09 h–l
16	Baimu 371	45.31 ± 0.93 k–m	23.40 ± 0.48 l–p	60.97 ± 1.01 tu	29.84 ± 1.25 e–h	44.86 ± 1.83 e–g	19.68 ± 1.18 i–m
17	Baimu 341	44.19 ± 0.70 l–n	25.62 ± 0.77 i–m	86.89 ± 0.25 bc	35.88 ± 0.43 cd	32.47 ± 0.47 i–l	26.48 ± 0.28 c–g
18	Knight 2	49.71 ± 0.34 i–l	32.43 ± 0.45 c–g	75.03 ± 0.72 m–q	28.84 ± 0.48 e–i	44.00 ± 1.31 e–g	29.82 ± 0.60 bc
19	Knight T	52.47 ± 2.03 g–k	33.63 ± 0.73 c–e	77.15 ± 0.98 i–p	30.13 ± 0.54 e–h	36.36 ± 1.74 h–j	26.49 ± 0.89 c–g
20	Milky way	51.48 ± 1.89 h–k	13.78 ± 0.36 st	86.00 ± 1.08 b–d	18.54 ± 0.94 lm	32.11 ± 1.71 i–l	17.53 ± 0.53 j–n
21	Greensilk	48.15 ± 1.53 j–l	38.98 ± 0.67 b	73.42 ± 0.83 n–q	30.91 ± 1.38 d–h	55.87 ± 1.91 bc	14.72 ± 0.28 n–p
22	Challenger	58.24 ± 1.46 e–h	35.54 ± 1.32 b–d	83.89 ± 0.33 c–g	27.45 ± 0.33 f–k	47.68 ± 1.93 d–f	32.89 ± 0.75 ab
23	Adina	72.32 ± 0.80 ab	15.37 ± 1.19 st	76.75 ± 0.98 j–p	24.29 ± 0.87 i–k	60.05 ± 2.37 b	9.90 ± 0.25 q
24	Conthey	66.89 ± 1.29 bc	26.74 ± 0.77 i–l	70.54 ± 0.39 qr	24.42 ± 0.94 i–k	66.99 ± 1.60 a	17.08 ± 0.49 k–o
25	Baimu 202	48.05 ± 1.17 j–l	24.66 ± 0.29 j–o	77.98 ± 1.53 h–n	33.83 ± 1.35 c–e	32.94 ± 0.89 i–l	22.36 ± 0.63 f–j
26	Baimu 401	61.37 ± 1.33 c–f	16.95 ± 0.90 q–s	74.94 ± 1.30 m–q	17.54 ± 1.03 m	44.39 ± 0.47 e–g	14.68 ± 0.32 n–p
27	Tecarat	65.78 ± 2.03 b–d	24.75 ± 0.64 j–o	73.98 ± 0.88 n–q	23.22 ± 0.82 kl	47.53 ± 0.58 d–f	18.81 ± 0.51 i–n
28	WL656HQ	71.20 ± 1.81 ab	36.16 ± 1.01 b–d	78.68 ± 0.37 h–m	36.75 ± 0.67 c	49.41 ± 1.53 c–f	19.70 ± 0.87 i–m
29	WL358HQ	55.34 ± 1.51 e–j	33.29 ± 1.09 c–f	77.87 ± 0.96 h–o	17.97 ± 0.43 m	35.16 ± 1.31 ij	12.56 ± 1.08 o–q
30	WL440HQ	74.73 ± 1.71 a	25.00 ± 1.11 i–n	80.21 ± 0.26 g–k	23.19 ± 1.06 kl	44.69 ± 0.21 e–g	19.03 ± 1.04 i–n
31	WL168HQ	51.24 ± 1.12 h–l	32.58 ± 0.87 c–g	81.22 ± 1.44 f–j	23.66 ± 0.81 i–k	33.60 ± 0.61 i–k	24.82 ± 0.69 d–h
32	WL343HQ	62.23 ± 2.26 c–e	26.30 ± 1.03 i–l	76.35 ± 0.64 k–p	28.61 ± 0.86 e–j	45.05 ± 1.77 e–g	21.08 ± 1.03 h–l
33	WL525HQ	55.02 ± 0.64 e–j	14.06 ± 0.44 st	88.87 ± 0.42 b	14.54 ± 0.58 m	43.28 ± 2.51 f–h	12.43 ± 0.73 pq
34	WL298HQ	70.55 ± 1.77 ab	29.33 ± 0.28 f–i	81.82 ± 0.87 d–h	31.85 ± 0.67 c–g	31.80 ± 1.63 i–l	29.30 ± 0.49 b–d
35	Zhongmu No. 1	55.14 ± 0.70 e–j	20.74 ± 0.72 n–q	101.89 ± 1.35 a	28.63 ± 0.18 e–j	51.70 ± 1.35 c–e	31.04 ± 1.24 bc
36	Gongnong No. 1	55.35 ± 1.39 e–j	12.47 ± 0.56 t	79.85 ± 0.60 g–l	17.83 ± 0.80 m	33.42 ± 2.56 i–l	12.88 ± 1.16 o–q
37	Zhaodong	55.21 ± 0.99 e–j	23.45 ± 0.60 l–p	75.59 ± 0.44 l–p	17.46 ± 1.16 m	30.86 ± 1.39 j–m	21.22 ± 1.16 h–l
38	Legacy	49.28 ± 1.39 i–l	28.51 ± 0.56 g–k	81.45 ± 0.32 e–i	32.93 ± 0.46 c–e	33.18 ± 1.27 i–l	27.16 ± 1.07 c–e
39	Canon	66.81 ± 1.80 bc	20.56 ± 0.59 o–q	59.69 ± 0.44 u	14.91 ± 0.78 m	23.85 ± 1.83 m	16.17 ± 1.01 m–p
40	Jiasheng	56.48 ± 1.75 e–i	16.26 ± 1.28 r–t	65.81 ± 0.87 s	25.66 ± 1.11 h–k	39.05 ± 0.12 g–i	18.52 ± 0.97 i–n

**Table 6:** Soluble protein content in roots of different varieties of alfalfa (mg/g).

No.	Variety	Soluble Protein					
		Taproot		Lateral Root		Root Crown	
		Before Overwintering	After Overwintering	Before Overwintering	After Overwintering	Before Overwintering	After Overwintering
1	Juneng 401	22.38 ± 0.19 c–g	14.84 ± 0.41 a–e	23.80 ± 0.95 b–e	10.25 ± 0.52 a–h	15.83 ± 0.20 d–g	8.78 ± 0.68 a–h
2	3010	22.67 ± 1.10 c–f	16.14 ± 0.78 a–d	23.61 ± 1.05 b–f	7.42 ± 0.49 f–i	16.38 ± 0.99 d–g	5.88 ± 0.81 e–h
3	4030	16.52 ± 0.12 i–l	15.29 ± 0.44 a–e	20.66 ± 0.84 c–m	10.93 ± 0.67 a–g	18.66 ± 0.64 b–e	6.50 ± 1.10 d–h
4	Juneng S.R	22.67 ± 1.11 c–f	16.08 ± 0.28 a–d	20.81 ± 1.34 c–l	8.08 ± 0.97 d–i	17.48 ± 0.77 c–f	8.77 ± 0.96 a–h
5	Juneng 995	14.31 ± 0.24 j–l	13.09 ± 0.46 b–g	14.37 ± 1.02 no	8.41 ± 0.41 d–i	14.21 ± 1.12 f–j	5.88 ± 0.52 e–h
6	4020	18.05 ± 0.45 g–k	13.02 ± 0.76 b–h	14.16 ± 1.13 o	7.96 ± 0.30 d–i	16.38 ± 0.75 d–g	7.97 ± 1.14 b–h
7	Tango	13.37 ± 0.57 l	15.07 ± 0.85 a–e	18.81 ± 0.64 e–o	5.80 ± 0.33 i	16.18 ± 0.53 d–g	9.92 ± 0.72 a–e
8	Fertress	15.10 ± 0.25 j–l	15.84 ± 0.13 a–d	20.03 ± 1.41 c–m	8.54 ± 0.63 c–i	14.53 ± 1.00 f–j	6.10 ± 0.81 d–h
9	Spider	13.52 ± 0.81 l	11.81 ± 1.04 d–j	17.47 ± 0.88 g–o	9.89 ± 0.29 b–h	11.52 ± 1.00 h–j	9.01 ± 0.81 a–h
10	Gladiator	21.03 ± 1.21 c–h	17.12 ± 0.47 ab	19.85 ± 0.35 c–n	7.39 ± 0.99 g–i	10.84 ± 0.94 j	9.40 ± 0.84 a–g
11	Juneng 7	20.16 ± 0.83 d–i	17.36 ± 0.82 ab	15.24 ± 0.46 l–o	7.70 ± 0.37 e–i	13.48 ± 0.47 g–j	10.13 ± 0.73 a–d
12	Juneng 801	21.95 ± 1.18 c–h	16.14 ± 0.60 a–d	14.45 ± 0.82 no	10.95 ± 1.26 a–g	14.18 ± 0.89 f–j	10.02 ± 0.76 a–e
13	Juneng 601	21.99 ± 1.24 c–h	16.85 ± 1.27 a–c	20.10 ± 1.18 c–m	11.62 ± 0.93 a–e	10.94 ± 0.52 ij	11.34 ± 0.79 ab
14	4010	21.42 ± 0.58 c–h	12.46 ± 1.01 c–i	24.06 ± 0.20 b–e	8.89 ± 0.57 b–i	10.79 ± 0.51 j	6.80 ± 0.72 c–h
15	Quattro	17.76 ± 0.30 h–k	12.14 ± 0.25 d–j	21.41 ± 0.38 b–j	7.99 ± 0.64 d–i	11.71 ± 1.09 h–j	9.94 ± 0.88 a–e
16	Baimu 371	18.78 ± 0.53 f–j	13.41 ± 0.32 a–g	19.29 ± 0.85 d–o	9.33 ± 0.46 b–i	13.33 ± 0.93 g–j	8.18 ± 0.48 b–h
17	Baimu 341	24.86 ± 1.13 bc	13.53 ± 0.71 a–f	23.97 ± 0.95 b–e	8.62 ± 0.37 b–i	19.48 ± 0.37 b–d	10.94 ± 0.54 a–c
18	Knight 2	21.25 ± 0.87 c–h	17.92 ± 0.89 a	20.19 ± 1.12 c–m	9.01 ± 0.91 b–i	18.65 ± 0.32 b–e	12.60 ± 0.89 a
19	Knight T	23.87 ± 0.71 b–d	17.15 ± 1.19 ab	15.15 ± 0.69 m–o	9.12 ± 0.54 b–i	16.85 ± 0.64 c–g	8.14 ± 0.18 b–h
20	Milky way	22.46 ± 0.91 c–g	11.87 ± 0.82 d–j	15.65 ± 0.81 k–o	9.66 ± 0.97 b–i	18.67 ± 0.64 b–e	7.61 ± 0.43 b–h
21	Greensilk	22.34 ± 1.40 c–g	10.81 ± 0.26 e–k	17.95 ± 1.01 f–o	8.98 ± 0.79 b–i	11.91 ± 0.77 h–j	7.24 ± 0.84 b–h
22	Challenger	22.48 ± 0.81 c–g	12.05 ± 0.50 d–j	21.15 ± 0.50 c–k	11.95 ± 0.17 a–d	13.82 ± 0.41 f–j	7.99 ± 0.92 b–h
23	Adina	20.10 ± 0.79 d–i	5.91 ± 2.95 l	22.51 ± 1.12 b–i	9.46 ± 0.80 b–i	13.95 ± 0.27 f–j	7.43 ± 0.81 b–h
24	Conthey	21.93 ± 1.04 c–h	9.40 ± 1.00 f–l	20.19 ± 0.63 c–m	11.50 ± 1.00 a–f	13.69 ± 0.22 f–j	8.90 ± 0.67 a–h
25	Baimu 202	20.48 ± 0.50 c–i	10.21 ± 0.27 f–l	15.26 ± 0.50 l–o	11.62 ± 0.68 a–e	13.77 ± 0.71 f–j	7.31 ± 0.78 b–h
26	Baimu 401	14.33 ± 0.88 j–l	6.96 ± 0.84 kl	22.74 ± 0.52 b–h	9.40 ± 0.51 b–i	15.27 ± 0.51 e–h	5.32 ± 0.31 gh
27	Tecarat	19.18 ± 0.66 e–j	7.71 ± 0.29 j–l	16.89 ± 0.96 i–o	8.89 ± 0.87 b–i	13.89 ± 0.23 f–j	6.86 ± 0.34 c–h
28	WL656HQ	19.19 ± 1.34 e–j	12.47 ± 0.80 c–i	17.18 ± 1.00 h–o	11.04 ± 0.71 a–g	14.11 ± 0.82 f–j	7.84 ± 0.93 b–h
29	WL358HQ	24.05 ± 0.32 b–d	8.29 ± 0.67 i–l	26.79 ± 1.50 b	6.48 ± 0.28 hi	13.63 ± 0.71 f–j	5.63 ± 0.55 f–h
30	WL440HQ	22.04 ± 1.01 c–h	8.88 ± 0.82 g–l	24.61 ± 0.96 b–d	10.15 ± 0.72 a–h	14.65 ± 0.39 f–j	8.35 ± 1.17 b–h
31	WL168HQ	27.15 ± 0.43 ab	8.30 ± 0.52 i–l	35.62 ± 1.37 a	11.81 ± 0.23 a–d	26.39 ± 0.28 a	9.67 ± 0.92 a–f
32	WL343HQ	24.02 ± 0.76 b–d	7.56 ± 0.76 j–l	22.26 ± 1.18 b–i	10.92 ± 1.39 a–g	16.56 ± 0.65 d–g	7.53 ± 0.56 b–h
33	WL525HQ	18.45 ± 0.98 f–j	9.25 ± 0.75 f–l	23.36 ± 1.72 b–f	9.40 ± 0.35 b–i	14.65 ± 1.04 f–j	10.00 ± 0.69 a–e
34	WL298HQ	29.54 ± 0.86 a	9.60 ± 0.96 f–l	25.55 ± 0.80 bc	9.14 ± 0.79 b–i	16.01 ± 0.54 d–g	11.35 ± 0.92 ab
35	Zhongmu No. 1	21.72 ± 0.53 c–h	8.32 ± 0.78 i–l	20.92 ± 1.50 c–k	12.70 ± 1.19 ab	20.31 ± 1.10 bc	6.07 ± 0.23 d–h
36	Gongnong No. 1	21.30 ± 0.38 c–h	7.09 ± 0.27 kl	22.90 ± 1.07 b–g	11.36 ± 0.94 a–g	21.45 ± 1.03 b	6.37 ± 0.62 d–h
37	Zhaodong	23.52 ± 0.58 b–e	7.63 ± 0.91 j–l	19.58 ± 1.57 d–o	9.74 ± 0.64 b–i	14.38 ± 0.34 f–j	5.07 ± 0.37 h
38	Legacy	21.87 ± 0.89 c–h	7.68 ± 0.77 j–l	22.36 ± 1.52 b–i	14.04 ± 0.92 a	14.81 ± 0.68 f–i	7.27 ± 0.88 b–h
39	Canon	22.85 ± 0.40 c–f	7.79 ± 0.70 j–l	15.97 ± 0.42 j–o	9.15 ± 0.45 b–i	14.30 ± 0.33 f–j	5.90 ± 0.69 e–h
40	Jiasheng	22.67 ± 0.76 c–f	8.47 ± 1.07 h–l	21.16 ± 1.59 c–k	12.59 ± 0.69 a–c	14.43 ± 0.77 f–j	7.01 ± 0.56 c–h

**Table 7:** C, N, and P content in roots of different varieties of alfalfa (%).

No.	Variety	C		N		P	
		Before Overwintering	After Overwintering	Before Overwintering	After Overwintering	Before Overwintering	After Overwintering
1	Juneng 401	46.73 ± 2.38 b–i	51.62 ± 0.59 b–e	7.02 ± 0.24 a–d	7.11 ± 0.14 a	0.26 ± 0.01 d–g	0.23 ± 0.00 i–m
2	3010	50.17 ± 1.21 a–e	48.74 ± 0.62 c–k	6.24 ± 0.36 a–d	6.51 ± 0.12 a–c	0.27 ± 0.01 d–f	0.20 ± 0.01 k–n
3	4030	47.77 ± 1.48 b–h	47.65 ± 0.96 c–l	6.30 ± 0.26 a–d	5.37 ± 0.16 a–e	0.31 ± 0.02 b–e	0.24 ± 0.01 g–k
4	Juneng S.R	56.08 ± 0.14 a	58.88 ± 0.90 a	5.92 ± 0.11 a–d	5.54 ± 0.50 a–e	0.27 ± 0.01 d–g	0.23 ± 0.00 h–l
5	Juneng 995	51.91 ± 0.93 a–d	55.35 ± 0.49 ab	7.33 ± 0.31 a–c	5.58 ± 0.49 a–e	0.20 ± 0.00 g	0.19 ± 0.01 mn
6	4020	52.35 ± 2.98 a–c	50.39 ± 1.29 b–g	7.05 ± 0.41 a–d	5.33 ± 0.74 a–e	0.32 ± 0.01 b–d	0.19 ± 0.01 n
7	Tango	52.67 ± 0.52 ab	53.52 ± 1.16 bc	7.07 ± 0.44 a–d	5.49 ± 0.29 a–e	0.30 ± 0.01 b–f	0.23 ± 0.01 i–m
8	Fertress	49.11 ± 1.10 a–f	48.73 ± 0.61 c–k	6.69 ± 0.28 a–d	6.27 ± 0.11 a–d	0.31 ± 0.02 b–d	0.28 ± 0.01 c–g
9	Spider	45.32 ± 2.46 b–i	47.60 ± 1.02 c–l	6.36 ± 0.37 a–d	6.03 ± 0.08 a–e	0.27 ± 0.01 d–f	0.23 ± 0.01 i–m
10	Gladiator	44.39 ± 1.02 d–k	44.61 ± 0.20 g–l	6.58 ± 0.42 a–d	5.70 ± 0.62 a–e	0.37 ± 0.03 ab	0.18 ± 0.01 n
11	Juneng 7	46.79 ± 1.97 b–i	52.86 ± 0.42 b–d	6.80 ± 0.33 a–d	5.86 ± 0.37 a–e	0.23 ± 0.00 e–g	0.23 ± 0.01 h–m
12	Juneng 801	40.99 ± 1.64 g–l	48.89 ± 0.57 c–k	6.55 ± 0.57 a–d	5.57 ± 0.40 a–e	0.23 ± 0.01 e–g	0.23 ± 0.01 h–l
13	Juneng 601	42.15 ± 0.38 f–k	46.94 ± 0.52 d–l	7.04 ± 0.73 a–d	6.47 ± 0.42 a–c	0.29 ± 0.03 c–f	0.25 ± 0.00 g–j
14	4010	46.02 ± 1.84 b–i	48.87 ± 0.16 c–k	7.60 ± 0.69 a	5.88 ± 0.63 a–e	0.32 ± 0.01 b–d	0.26 ± 0.00 e–i
15	Quattro	44.76 ± 1.61 c–j	49.87 ± 0.99 b–i	6.34 ± 0.38 a–d	5.97 ± 0.17 a–e	0.30 ± 0.01 b–f	0.21 ± 0.00 j–n
16	Baimu 371	43.14 ± 2.02 e–k	49.38 ± 0.55 c–j	5.52 ± 0.03 a–d	6.05 ± 0.49 a–e	0.29 ± 0.01 b–f	0.24 ± 0.01 h–l
17	Baimu 341	44.82 ± 1.21 c–j	48.28 ± 1.34 c–k	5.96 ± 0.36 a–d	6.03 ± 0.18 a–e	0.31 ± 0.01 b–e	0.20 ± 0.01 k–n
18	Knight 2	45.27 ± 0.29 b–i	46.51 ± 1.55 d–l	5.71 ± 0.61 a–d	6.00 ± 0.49 a–e	0.29 ± 0.00 c–f	0.25 ± 0.00 f–i
19	Knight T	48.80 ± 1.09 b–f	46.08 ± 0.55 e–l	6.43 ± 0.16 a–d	4.35 ± 0.30 c–e	0.29 ± 0.01 c–f	0.25 ± 0.01 f–i
20	Milky way	44.38 ± 1.57 d–k	47.35 ± 1.03 c–l	6.05 ± 0.27 a–d	4.32 ± 0.26 c–e	0.27 ± 0.01 d–f	0.21 ± 0.01 j–n
21	Greensilk	44.48 ± 0.48 d–k	50.27 ± 0.78 b–h	6.07 ± 0.20 a–d	4.06 ± 0.30 e	0.26 ± 0.00 d–g	0.23 ± 0.01 h–m
22	Challenger	42.75 ± 0.75 e–k	51.20 ± 1.12 b–f	6.09 ± 0.79 a–d	4.46 ± 0.23 b–e	0.27 ± 0.01 d–f	0.24 ± 0.01 h–l
23	Adina	40.56 ± 1.71 g–m	47.43 ± 1.28 c–l	6.34 ± 0.14 a–d	4.17 ± 0.14 de	0.31 ± 0.01 b–d	0.20 ± 0.01 k–n
24	Conthey	39.95 ± 1.18 i–m	48.66 ± 1.44 c–k	5.46 ± 0.24 b–d	4.37 ± 0.11 b–e	0.31 ± 0.01 b–d	0.26 ± 0.01 f–i
25	Baimu 202	48.91 ± 1.11 b–f	44.39 ± 0.90 g–l	5.39 ± 0.34 cd	7.19 ± 0.31 a	0.30 ± 0.01 b–f	0.29 ± 0.01 b–f
26	Baimu 401	43.46 ± 0.55 e–k	43.29 ± 1.02 j–l	5.71 ± 0.06 a–d	6.00 ± 0.54 a–e	0.28 ± 0.01 c–f	0.25 ± 0.00 f–i
27	Tecarat	40.24 ± 1.91 h–m	46.78 ± 1.66 d–l	6.49 ± 0.25 a–d	7.01 ± 0.25 a	0.22 ± 0.03 fg	0.26 ± 0.00 f–i
28	WL656HQ	42.02 ± 1.57 f–k	48.26 ± 2.03 c–k	5.59 ± 0.42 a–d	6.96 ± 0.30 a	0.22 ± 0.03 fg	0.20 ± 0.00 k–n
29	WL358HQ	50.07 ± 2.12 a–e	50.40 ± 1.51 b–g	6.22 ± 0.32 a–d	6.89 ± 0.04 a	0.30 ± 0.03 b–f	0.20 ± 0.00 k–n
30	WL440HQ	47.63 ± 1.41 b–h	48.52 ± 0.95 c–k	5.04 ± 0.48 d	7.04 ± 0.39 a	0.22 ± 0.02 fg	0.24 ± 0.01 h–l
31	WL168HQ	32.59 ± 0.83 n	41.92 ± 1.89 l	5.45 ± 0.19 b–d	6.94 ± 0.27 a	0.33 ± 0.01 b–d	0.32 ± 0.01 b
32	WL343HQ	34.97 ± 0.26 l–n	43.59 ± 1.99 i–l	5.39 ± 0.16 cd	7.35 ± 0.82 a	0.28 ± 0.03 c–f	0.20 ± 0.00 l–n
33	WL525HQ	37.65 ± 1.04 j–n	48.42 ± 0.90 c–k	6.46 ± 0.45 a–d	6.81 ± 0.14 a	0.30 ± 0.01 b–f	0.23 ± 0.00 h–m
34	WL298HQ	33.79 ± 0.82 mn	47.69 ± 0.56 c–l	6.22 ± 0.14 a–d	6.74 ± 0.52 a	0.30 ± 0.01 b–f	0.27 ± 0.01 d–h
35	Zhongmu No. 1	37.14 ± 1.23 k–n	45.19 ± 2.20 f–l	6.77 ± 0.40 a–d	6.33 ± 0.22 a–d	0.32 ± 0.02 b–d	0.24 ± 0.01 g–k
36	Gongnong No. 1	50.09 ± 0.23 a–e	47.86 ± 1.27 c–l	6.05 ± 0.46 a–d	6.21 ± 0.01 a–e	0.28 ± 0.02 c–f	0.36 ± 0.00 a
37	Zhaodong	46.48 ± 1.47 b–i	42.55 ± 1.01 kl	6.53 ± 0.42 a–d	6.55 ± 0.22 ab	0.42 ± 0.01 a	0.30 ± 0.01 b–e
38	Legacy	48.70 ± 1.03 b–f	43.89 ± 0.75 h–l	7.61 ± 0.15 a	5.81 ± 0.44 a–e	0.33 ± 0.01 b–d	0.30 ± 0.01 b–d
39	Canon	48.03 ± 0.84 b–g	48.02 ± 1.73 c–l	7.57 ± 0.35 ab	6.36 ± 0.63 a–c	0.33 ± 0.00 b–d	0.29 ± 0.00 b–f
40	Jiasheng	48.21 ± 0.98 b–g	45.71 ± 0.56 e–l	5.99 ± 0.14 a–d	7.00 ± 0.69 a	0.36 ± 0.01 a–c	0.31 ± 0.00 bc

**Table 8:** Stoichiometric characteristics of C, N, and P in roots of different alfalfa varieties.

No.	Variety	C/N		N/P		C/P	
		Before Overwintering	After Overwintering	Before Overwintering	After Overwintering	Before Overwintering	After Overwintering
1	Juneng 401	6.67 ± 0.29 c–e	7.26 ± 0.08 g–i	27.13 ± 1.91 b–d	31.38 ± 0.82 a–e	180.55 ± 13.57 b–f	222.87 ± 5.37 b–h
2	3010	8.08 ± 0.30 a–e	7.49 ± 0.08 g–i	22.95 ± 2.04 b–f	32.55 ± 0.37 a–d	184.16 ± 9.87 b–e	242.97 ± 4.58 b–d
3	4030	7.62 ± 0.51 a–e	8.87 ± 0.15 b–i	20.88 ± 2.31 b–f	22.42 ± 0.51 d–h	157.30 ± 10.82 c–l	192.07 ± 7.61 d–l
4	Juneng S.R	9.48 ± 0.19 ab	10.84 ± 1.19 a–e	21.97 ± 0.75 b–f	23.73 ± 2.07 b–h	207.92 ± 4.81 bc	252.53 ± 7.27 a–c
5	Juneng 995	7.10 ± 0.25 a–e	10.05 ± 0.76 a–g	37.31 ± 1.88 a	29.43 ± 2.68 a–f	264.26 ± 9.28 a	292.98 ± 10.71 a
6	4020	7.53 ± 0.85 a–e	9.76 ± 1.13 a–h	22.28 ± 1.16 b–f	29.08 ± 5.52 a–f	166.55 ± 15.76 b–j	272.57 ± 17.81 ab
7	Tango	7.52 ± 0.55 a–e	9.79 ± 0.43 a–h	23.26 ± 1.03 b–f	24.46 ± 2.42 b–h	173.88 ± 5.58 b–h	232.37 ± 13.29 b–f
8	Fertress	7.37 ± 0.39 a–e	7.78 ± 0.22 e–i	21.60 ± 0.16 b–f	22.70 ± 0.82 c–h	159.26 ± 9.26 c–l	172.40 ± 4.48 i–n
9	Spider	7.20 ± 0.73 a–e	7.89 ± 0.15 e–i	23.22 ± 0.84 b–f	26.71 ± 1.17 a–h	166.32 ± 12.38 b–j	212.00 ± 12.83 c–k
10	Gladiator	6.81 ± 0.53 b–e	7.99 ± 0.78 e–i	18.11 ± 2.21 d–f	32.48 ± 4.21 a–d	121.03 ± 6.56 i–m	252.27 ± 10.29 a–c
11	Juneng 7	6.92 ± 0.52 a–e	9.09 ± 0.56 b–i	29.97 ± 1.05 ab	25.63 ± 2.13 b–h	206.53 ± 9.39 bc	232.15 ± 13.42 b–h
12	Juneng 801	6.35 ± 0.59 de	8.85 ± 0.53 b–i	28.82 ± 1.62 bc	24.03 ± 2.45 b–h	181.12 ± 7.31 b–f	212.02 ± 8.39 c–k
13	Juneng 601	6.12 ± 0.62 de	7.32 ± 0.46 g–i	24.79 ± 2.31 b–f	26.20 ± 1.44 b–h	150.59 ± 17.97 d–l	192.33 ± 2.05 e–m
14	4010	6.11 ± 0.32 de	8.50 ± 0.87 c–i	23.93 ± 1.39 b–f	22.62 ± 2.41 c–h	145.38 ± 3.25 d–m	182.95 ± 0.60 f–m
15	Quattro	7.10 ± 0.39 a–e	8.37 ± 0.38 d–i	21.32 ± 0.77 b–f	28.90 ± 0.90 a–g	150.91 ± 5.14 d–l	242.32 ± 3.78 b–d
16	Baimu 371	7.81 ± 0.35 a–e	8.25 ± 0.57 d–i	18.85 ± 0.58 d–f	25.64 ± 2.42 b–h	147.73 ± 11.14 d–m	202.07 ± 7.68 c–k
17	Baimu 341	7.58 ± 0.53 a–e	8.01 ± 0.21 e–i	19.39 ± 0.73 c–f	30.30 ± 1.75 a–f	146.35 ± 5.76 d–m	242.05 ± 17.18 b–d
18	Knight 2	8.13 ± 0.93 a–e	7.86 ± 0.74 e–i	19.94 ± 2.26 c–f	24.01 ± 1.95 b–h	157.95 ± 2.08 c–l	182.05 ± 6.20 g–m
19	Knight T	7.60 ± 0.21 a–e	10.70 ± 0.82 a–f	22.20 ± 0.78 b–f	17.45 ± 1.28 h	168.28 ± 2.14 b–j	182.15 ± 9.45 g–m
20	Milky way	7.35 ± 0.19 a–e	11.04 ± 0.79 a–d	22.18 ± 1.30 b–f	21.09 ± 2.16 d–h	162.81 ± 9.64 c–k	222.99 ± 11.37 b–h
21	Greensilk	7.35 ± 0.33 a–e	12.50 ± 0.81 a	23.62 ± 0.50 b–f	17.61 ± 0.94 gh	173.41 ± 4.15 b–h	212.70 ± 3.60 c–j
22	Challenger	7.21 ± 0.76 a–e	11.53 ± 0.57 ab	22.46 ± 3.51 b–f	19.02 ± 1.76 f–h	156.71 ± 6.57 c–l	212.31 ± 10.68 c–j
23	Adina	6.40 ± 0.28 c–e	11.42 ± 0.67 a–c	20.46 ± 0.40 b–f	20.53 ± 0.67 e–h	131.08 ± 7.60 f–m	232.00 ± 12.55 b–g
24	Conthey	7.34 ± 0.31 a–e	11.15 ± 0.51 a–d	17.64 ± 1.00 d–f	17.05 ± 0.28 h	129.39 ± 8.16 f–m	192.35 ± 11.78 e–m
25	Baimu 202	9.13 ± 0.43 a–c	6.20 ± 0.30 i	17.96 ± 1.05 d–f	25.07 ± 0.39 b–h	163.20 ± 5.45 c–k	152.18 ± 6.12 l–n
26	Baimu 401	7.62 ± 0.16 a–e	7.33 ± 0.65 g–i	20.18 ± 0.67 c–f	23.69 ± 2.22 b–h	153.57 ± 3.13 d–l	172.03 ± 6.19 j–n
27	Tecarat	6.19 ± 0.09 de	6.69 ± 0.32 hi	29.76 ± 2.84 ab	27.34 ± 1.15 a–h	183.98 ± 15.54 b–e	182.47 ± 8.83 h–m
28	WL656HQ	7.62 ± 0.75 a–e	6.93 ± 0.15 g–i	26.17 ± 5.00 b–e	34.31 ± 1.98 ab	192.13 ± 18.11 b–d	232.79 ± 13.66 b–e
29	WL358HQ	8.12 ± 0.72 a–e	7.31 ± 0.24 g–i	21.04 ± 1.89 b–f	33.92 ± 0.71 a–c	170.13 ± 19.90 b–i	242.82 ± 4.61 a–d
30	WL440HQ	9.59 ± 0.79 a	6.93 ± 0.34 g–i	22.64 ± 1.68 b–f	29.80 ± 1.85 a–f	215.03 ± 11.28 b	202.32 ± 4.68 c–k
31	WL168HQ	6.00 ± 0.32 de	6.05 ± 0.31 i	16.56 ± 1.01 ef	21.92 ± 0.16 d–h	98.79 ± 1.48 m	132.54 ± 5.95 n
32	WL343HQ	6.50 ± 0.20 c–e	6.10 ± 0.79 i	19.60 ± 2.50 c–f	37.55 ± 4.86 a	126.62 ± 13.69 g–m	222.50 ± 7.78 c–i
33	WL525HQ	5.89 ± 0.51 de	7.12 ± 0.15 g–i	21.66 ± 2.06 b–f	29.59 ± 0.60 a–f	125.57 ± 1.26 h–m	212.52 ± 3.89 c–k
34	WL298HQ	5.45 ± 0.26 e	7.14 ± 0.44 g–i	20.81 ± 1.16 b–f	24.96 ± 1.64 b–h	112.78 ± 2.44 k–m	172.02 ± 5.57 i–n
35	Zhongmu No. 1	5.54 ± 0.46 e	7.13 ± 0.16 g–i	21.45 ± 1.43 b–f	26.57 ± 2.10 b–h	117.67 ± 4.83 j–m	192.04 ± 18.32 e–m
36	Gongnong No. 1	8.39 ± 0.68 a–d	7.70 ± 0.19 f–i	21.30 ± 0.39 b–f	17.10 ± 0.15 h	178.10 ± 11.12 b–g	132.73 ± 3.25 n
37	Zhaodong	7.19 ± 0.58 a–e	6.51 ± 0.31 i	15.53 ± 0.87 f	22.11 ± 0.93 d–h	110.79 ± 4.76 lm	142.50 ± 2.09 mn
38	Legacy	6.40 ± 0.07 c–e	7.65 ± 0.65 f–i	22.86 ± 0.62 b–f	19.31 ± 0.79 f–h	146.35 ± 5.46 d–m	142.76 ± 7.10 mn
39	Canon	6.38 ± 0.35 de	7.70 ± 0.81 f–i	22.71 ± 1.14 b–f	22.14 ± 2.02 d–h	144.15 ± 3.58 d–m	162.67 ± 7.60 k–n
40	Jiasheng	8.05 ± 0.14 a–e	6.64 ± 0.55 hi	16.52 ± 0.68 ef	22.35 ± 2.32 d–h	132.80 ± 4.06 e–m	142.91 ± 1.90 mn

**Table 9:** Correlation analysis between winter survival rate and root physiology of alfalfa.

	WSR	FTSS	ATSS	FLSS	ALSS	FCSS	ACSS	FTST	ATST	FLST	ALST	FCST	ACST	FTSP	ATSP	FLSP	ALSP	FCSP	ACSP
WSR	1																		
FTSS	−0.04	1																	
ATSS	0.01	−0.07	1																
FLSS	0.08	0.02	0.01	1															
ALSS	0.09	0.01	0.35 **	0.10	1														
FCSS	−0.07	0.01	−0.03	0.11	0.15	1													
ACSS	0.05	−0.04	0.28 **	−0.24 **	0.18 *	−0.37 **	1												
FTST	−0.02 *	0.10	−0.02	0.25 **	0.03	0.47 **	−0.32 **	1											
ATST	0.01	−0.04	0.33 **	−0.12	0.30 **	−0.16	0.32 **	0.04	1										
FLST	−0.02	−0.08	0.18 *	0.10	0.28 **	0.15	0.15	0.10	0.02	1									
ALST	−0.04	−0.15	0.33 **	−0.31 **	0.25 **	−0.04	0.20 *	−0.21 *	0.30 **	−0.16	1								
FCST	−0.27 **	−0.05	0.28 **	−0.14	0.20 *	0.39 **	−0.09	0.27 **	0.21 *	0.06	0.16	1							
ACST	0.15	0.02	0.20*	−0.17	0.19 *	−0.20 *	0.64 **	−0.18 *	0.51 **	0.31 **	0.37 **	−0.13	1						
FTSP	0.03	0.07	0.01	0.25 **	0.03	0.08	−0.02	0.20 *	0.17	0.21 *	−0.22 *	−0.30 **	0.26 **	1					
ATSP	−0.12	−0.19 *	0.26 **	−0.32 **	−0.11	−0.46 **	0.57 **	−0.29 **	0.44 **	0.01	0.26 **	0.15	0.46 **	−0.16	1				
FLSP	0.06	−0.04	−0.05	0.18	−0.02	0.13	0.03	−0.04	−0.01	0.17	−0.29 **	−0.29 **	0.03	0.41 **	−0.31 **	1			
ALSP	−0.01	0.35 **	−0.11	0.17	0.23 *	0.30 **	−0.10	0.19 *	−0.15	0.30 **	−0.12	−0.02	0.13	0.22 *	−0.32 **	0.15	1		
FCSP	−0.04	0.20 *	0.01	−0.16	−0.11	0.01	0.01	−0.13	−0.21 *	0.23 *	−0.11	−0.19 *	0.09	0.29 **	−0.11	0.40 **	0.18	1	
ACSP	−0.02	−0.18	0.26 **	0.04	0.08	−0.01	0.38 **	−0.02	0.30 **	0.20 *	0.33 **	0.06	0.44 **	0.15	0.41 **	0.05	−0.07	0.02	1

WSR, winter survival rate; F, before overwintering; A, after overwintering; T, taproot; L, lateral root; C, root crown; SS, soluble sugar; ST, starch; SP, soluble protein. *, significant correlation at the *p* < 0.05 level; **, significant correlation at the *p* < 0.01 level.

**Table 10:** Correlation analysis between winter survival rate and root C, N, P contents and stoichiometric characteristics of alfalfa.

	WSR	FC	AC	FN	AN	FP	AP	FC/N	AC/N	FN/P	AN/P	FC/P	AC/P
WSR	1												
FC	−0.18	1											
AC	−0.38 **	0.42 **	1										
FN	0.02	0.21 *	0.18 *	1									
AN	0.15	−0.10	−0.20 *	−0.11	1								
FP	0.33 **	−0.02	−0.43 **	0.12	0.07	1							
AP	0.12	−0.02	−0.35 **	−0.04	0.19 *	0.29 **	1						
FC/N	−0.15	0.60 **	0.17	−0.64 **	0.02	−0.12	0.03	1					
AC/N	−0.25 **	0.20 *	0.49 **	0.11	−0.92 **	−0.20*	−0.28 **	0.05	1				
FN/P	−0.26 **	0.14	0.45 **	0.54 **	−0.08	−0.74 **	−0.27 **	−0.33 **	0.19 *	1			
AN/P	0.06	−0.05	0.08	−0.07	0.68 **	−0.12	−0.58 **	0.02	−0.55 **	0.11	1		
FC/P	−0.36 **	0.58 **	0.57 **	0.04	−0.07	−0.79 **	−0.24 **	0.42 **	0.23 *	0.71 **	0.11	1	
AC/P	−0.22 *	0.21 *	0.65 **	0.11	−0.20 *	−0.36 **	−0.91 **	0.06	0.39 **	0.38 **	0.52 **	0.42 **	1

WSR, winter survival rate; F, before overwintering; A, after overwintering; C, carbon; N, nitrogen; P, phosphorous. *, significant correlation at the *p* < 0.05 level; **, significant correlation at the *p* < 0.01 level.

**Table 11:** Comprehensive ranking of principal component analysis of different varieties of different alfalfa varieties.

Variety	Comprehensive Evaluation Value	Ranking	Variety	Comprehensive Evaluation Value	Ranking
Juneng 401	22.7084	37	Greensilk	30.6168	21
3010	17.3492	40	Challenger	34.1920	10
4030	29.2952	22	Adina	34.0476	11
Juneng S.R	30.6910	19	Conthey	35.0140	6
Juneng 995	22.6625	38	Baimu 202	36.5545	5
4020	23.0318	36	Baimu 401	32.4390	13
Tango	24.3813	34	Tecarat	31.8329	16
Fertress	32.3697	14	WL656HQ	30.9997	18
Spider	27.8453	26	WL358HQ	23.0995	35
Gladiator	29.0479	23	WL440HQ	30.6326	20
Juneng 7	34.2318	9	WL168HQ	37.5268	2
Juneng 801	34.4681	7	WL343HQ	28.2846	25
Juneng 601	27.3309	27	WL525HQ	24.6999	33
4010	28.4584	24	WL298HQ	32.6664	12
Quattro	21.6674	39	Zhongmu No. 1	37.2127	4
Baimu 371	26.8346	30	Gongnong No. 1	37.4803	3
Baimu 341	27.0557	29	Zhaodong	34.2596	8
Knight 2	31.6018	17	Legacy	38.1563	1
Knight T	26.2483	31	Canon	27.1048	28
Milky way	25.0183	32	Jiasheng	32.3398	15

**Table 12:** The score and rank of the comprehensive values calculated by the subordinate function analysis of the alfalfa varieties.

Variety	Comprehensive Evaluation Value	Ranking	Variety	Comprehensive Evaluation Value	Ranking
Juneng 401	0.62	31	Greensilk	0.64	26
3010	0.55	35	Challenger	0.66	20
4030	0.60	32	Adina	0.55	36
Juneng S.R	0.50	38	Conthey	0.53	37
Juneng 995	0.48	39	Baimu 202	0.75	6
4020	0.65	22	Baimu 401	0.71	11
Tango	0.63	30	Tecarat	0.69	17
Fertress	0.76	5	WL656HQ	0.55	34
Spider	0.69	16	WL358HQ	0.65	23
Gladiator	0.79	4	WL440HQ	0.58	33
Juneng 7	0.71	10	WL168HQ	0.71	8
Juneng 801	0.46	40	WL343HQ	0.80	3
Juneng 601	0.86	1	WL525HQ	0.63	29
4010	0.73	7	WL298HQ	0.67	19
Quattro	0.64	25	Zhongmu No. 1	0.70	13
Baimu 371	0.71	9	Gongnong No. 1	0.70	14
Baimu 341	0.67	18	Zhaodong	0.84	2
Knight 2	0.69	15	Legacy	0.71	12
Knight T	0.65	21	Canon	0.64	28
Milky way	0.64	27	Jiasheng	0.65	24

## Data Availability

The datasets generated during and/or analyzed during the current study are available from the corresponding author on reasonable request.
